# Three-Dimensional CH/π and CH/N Interactions from Quantum-Mechanical and Database Analyses

**DOI:** 10.1021/acs.jcim.5c00124

**Published:** 2025-04-14

**Authors:** Daichi Hayakawa, Hiroaki Gouda

**Affiliations:** † Division of Biophysical Chemistry, Department of Pharmaceutical Sciences, Graduate School of Pharmacy, Showa University, 1-5-8, Hatanodai, Shinagawa-ku, Tokyo, 142-8555, Japan

## Abstract

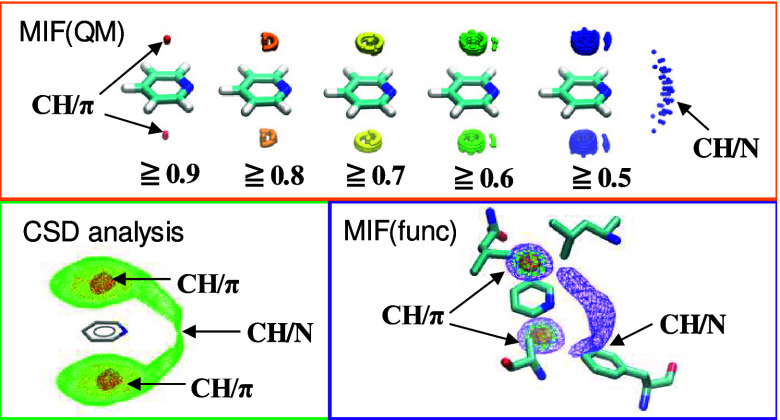

Quantum mechanical (QM)-level molecular interaction fields (MIFs) are three-dimensional potential maps that describe the intermolecular interactions surrounding a target molecule, derived through QM calculations. This study employs QM-level MIFs (MIFs(QM)) and analyses of the Cambridge Structural Database (CSD) to uncover the three-dimensional characteristics of CH/π and CH/N interactions in typical nitrogen-containing heterocyclic compounds. Our findings confirm the reliability and applicability of MIF(QM) calculations for analyzing CH/π and CH/N interactions. Additionally, we propose approximation functions of MIFs(QM) and demonstrate that the resulting MIFs(func) are effective for studying CH/π and CH/N interactions in protein/ligand systems.

## Introduction

Quantum mechanical (QM) calculations, such as molecular orbital theory and density functional theory (DFT) calculations, are essential tools for analyzing intermolecular interactions.^1^ To facilitate three-dimensional (3D) analyses of these interactions using QM calculations, we previously proposed a QM-level molecular interaction field (MIF) calculation.^2,3^ MIFs are 3D potential maps that describe intermolecular interactions surrounding a target molecule. They are generated by calculating the interactions between a target molecule and a probe molecule positioned at predefined grid points around the target molecule.^2−5^ MIFs capture the spatial characteristics of intermolecular interactions and have been widely applied in molecular modeling.^2−8^ One notable advantage of QM-level MIFs (MIFs(QM)) is their ability to accurately describe weak hydrogen bonds, which are challenging to represent using conventional force field-based MIFs.^2,3^

Weak hydrogen bonds, characterized by low interaction energies, include interactions such as CH/π and CH/N.^9−12^ The CH/π interaction is a weak hydrogen bond formed between a CH group, such as those in alkyl residues, and the π-electrons of aromatic groups.^9−12^ CH/N interactions occur between a CH group and the lone pair electrons of a nitrogen atom.^11,12^ According to previously published books,^10,11^ CH/π interactions were first recognized by Tamres in 1952,^13^ while CH/N interactions were identified by Kumler in 1935.^14^ Since their discovery, these interactions have been extensively studied through experimental methods such as infrared spectroscopy,^15^ nuclear magnetic resonance,^16^ and X-ray crystallography.^17,18^ Statistical analyses of the Cambridge Structural Database (CSD) in the 1980s and 1990s provided further evidence for their prevalence.^19−21^ Additionally, studies of Protein Data Bank (PDB)^22^ revealed that weak hydrogen bonds play significant roles in biological molecular systems, including proteins and nucleic acids.^23−25^ These interactions are therefore considered critical in structure-based drug design (SBDD).^26−30^ In the ligand binding sites of proteins, ligand molecules often form noncovalent bonds in multiple directions. The 3D features of weak hydrogen bonds, as revealed by MIF(QM) calculations, offer valuable insights for SBDD and understanding molecular recognition by proteins.

The primary limitation of MIF(QM) calculations is their difficult experimental validations, as MIFs(QM) cannot be directly observed through experiments. In our previous studies,^2,3^ we indirectly validated MIFs(QM) by comparing the calculated maps with X-ray crystal structures of protein/ligand systems. However, these validations were restricted to only certain aspects of the 3D features of MIFs(QM).

In the present work, we aim to address this limitation by combining MIFs(QM) with contour density maps obtained from CSD analyses to reveal the 3D features of CH/π and CH/N interactions.^31−33^ Contour density maps are 3D maps describing the number density of specific atoms of contact groups surrounding a target central atomic group. These maps are derived from statistical analyses of experimental crystal structures in the CSD and reflect the statistical probability of observing a contact group at a given point. For simplicity, we denote the contour density maps obtained by CSD analyses as density(CSD) maps in the present study. While MIF(QM) maps describe interaction energy, the density(CSD) maps describe statistical probability; thus, the two are complementary and comparable. Density(CSD) maps derived from experimental data validate the theoretically calculated MIFs(QM), while MIFs(QM) provide energetic explanations for the patterns observed in the density(CSD) maps. In this study, we selected aromatic nitrogen-containing heterocyclic compounds as model molecules. These compounds are common scaffolds in drugs and bioactive compounds and can form both CH/π and CH/N interactions, making them valuable targets for studying weak hydrogen bonds.

Here, we demonstrate that combining MIF(QM) and CSD analyses enables the elucidation of the 3D feature of CH/π and CH/N interactions. Through these analyses, we confirm the reliability and applicability of MIF(QM) calculations. Furthermore, we simplified the obtained MIFs using Gaussian expansions, resulting in approximation functions for MIFs(QM). We also present an example application of these approximation functions in analyzing weak hydrogen bonds in protein/ligand systems. The present study mainly focuses on the 3D understanding of the CH/π and CH/N interactions based on both the MIF(QM) and analyses of crystal data. To the best of our knowledge, similar studies have not been reported.

## Method

### Model Molecules

Table S1 lists the number of proteins that bind ligands with nitrogen-containing heterocyclic substructures registered in the PDB. Among double-ring nitrogen-containing heterocyclic compounds, ligands with purine substructures are the most common, followed by ligands with indole, quinoline, or benzimidazole substructures. Protein/indazole complex structures are also frequently observed. For single-ring nitrogen-containing heterocyclic compounds, ligands with pyrimidine substructures are the most prevalent, followed by those with imidazole, pyridine, or pyrrole substructures.

**Figure 1:**
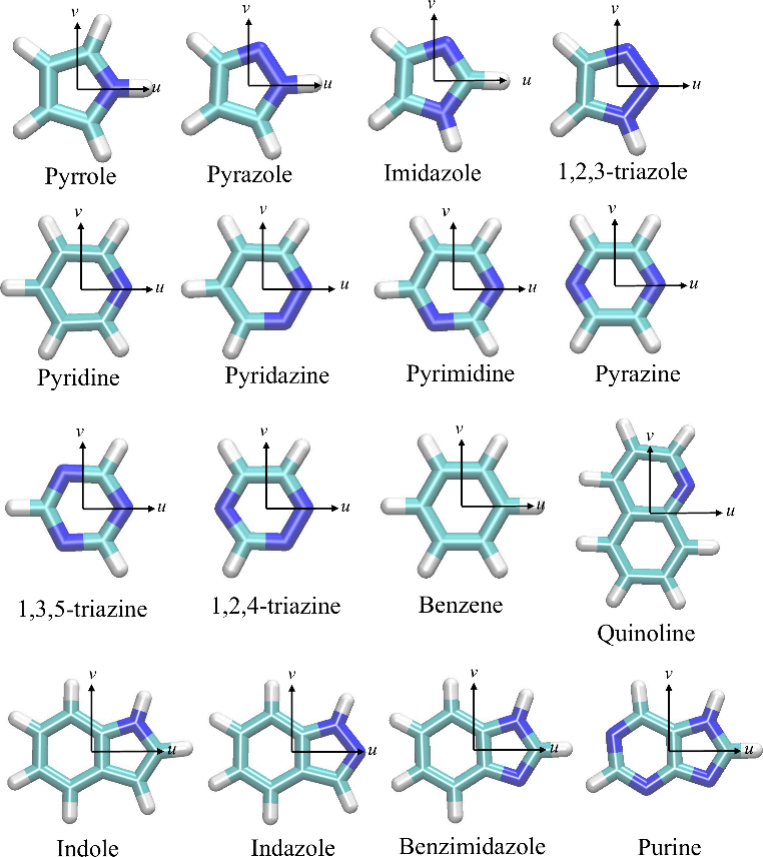
Sixteen compounds adopted as model molecules for MIF(QM) calculations. The *u* and *v* axes of the molecular coordinate system defined for each compound are shown.

The prevalence of these complex structures in the PDB highlights the widespread occurrence of these scaffolds in bioactive molecules. Therefore, we selected model molecules for MIFs(QM) calculations based on the frequency of the corresponding protein/ligand complexes in the PDB. For five-membered nitrogen-containing heterocyclic compounds, we used pyrrole, pyrazole, imidazole, and 1,2,3-triazole as model molecules. For six-membered nitrogen-containing heterocyclic compounds, pyridine, pyridazine, pyrimidine, pyrazine, 1,3,5-triazine, and 1,2,4-triazine were chosen. Additionally, five double-ring nitrogen-containing heterocycle compounds—quinoline, indole, indazole, benzimidazole, and purine—were included as model molecules. Benzene was also included as a reference molecule. In total, 16 compounds were selected as model molecules for MIF(QM) calculations (Figure 1).

### MIF(QM) Calculations

**Figure 2:**
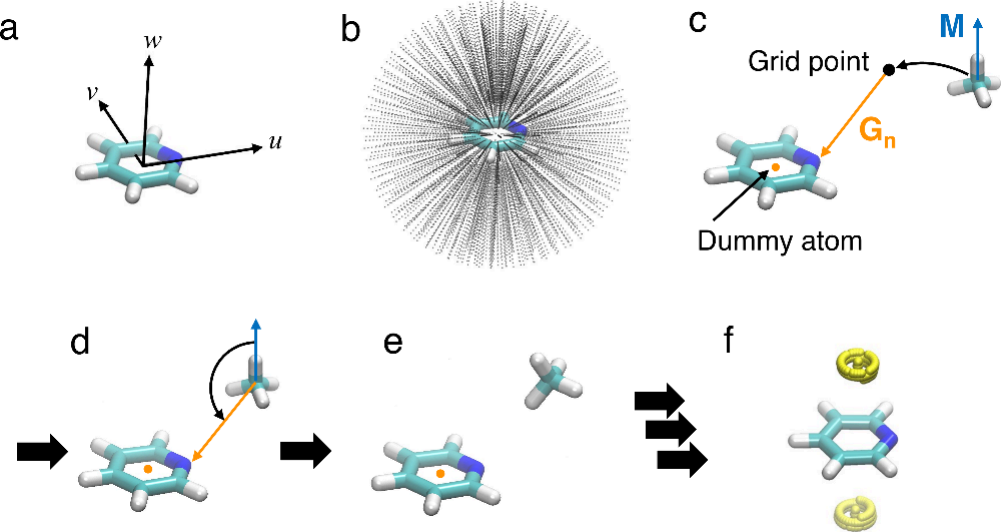
Procedure of MIF(QM) calculation: (a) Definition of the molecular coordinate system (*o-uvw*). (b) Definition of the grid points. (c) Positioning of the probe molecule at a grid point. (d) Rotation of the probe molecule to align with the target molecule. (e) Formation of the complex structure for calculating intermolecular interaction energy. (f) Visualization of the resulting MIF(QM) map. Grid points with MIF energy values of 0.7 or higher are displayed as yellow spheres as an example.

In this study, MIF(QM) calculations were performed following the procedure outlined in our previous works.^2,3^ The procedure is shown in Figure 2. A molecular coordinate system (*o-uvw*) is established for each target nitrogen-containing heterocyclic molecule, as shown in Figure 2a. The origin of the coordinate system is set at the center of the ring atoms, with the *w*-axis defined perpendicular to the ring plane. The *u*-axis is defined as extending from the center to a heavy atom within the target molecule, as specified in Figure 1. The *v*-axis is defined perpendicular to both the *u* and *w* axes. Spherical grid points are then arranged around the model molecule (Figure 2b). The grid points spanned radial distances from 2 to 7 Å at 0.2 Å intervals, with polar and azimuthal angles ranging from 0° to 180° and 0° to 360°, respectively, at 10° increments. This setup yielded a total of 15,964 grid points. A probe molecule was positioned at each grid point (Figure 2c). Methane, ethylene, benzene, and acetylene were used as probe molecules. For each probe molecule, a vector **M** was defined along the C–H axis, with its origin positioned at the carbon atom. A vector **G**_n_ was established from each grid point to the nearest atom of the target molecule. Additionally, dummy atoms were defined at the centers of aromatic rings and treated as equivalent to real atoms in defining the **G**_n_ vector. When a dummy atom was closest to a grid point, the **G**_n_ vector was defined from the grid point to the dummy atom. The probe molecule was then rotated such that vector **M** aligned with vector **G**_n_ (Figure 2d). The intermolecular interaction energy for this configuration was determined using QM calculations (Figure 2e). This process was repeated for all defined grid points. The resulting intermolecular interaction energies were normalized to values ranging from 0 to 1, based on the most stable energy value. We defined the normalized energies as the MIF(QM) energies at the respective grid points. The 3D map was generated based on the obtained MIF(QM) energies (Figure 2f) and referred to as the MIF(QM) maps. The MIFs(QM) calculated using methane, ethylene, benzene, and acetylene as probe molecules were denoted as MIF(QM, CH_4_), MIF(QM, CH_2_CH_2_), MIF(QM, BZ), and MIF(QM, CHCH), respectively. In regions with positive interaction energy values—indicative of steric repulsions—the MIF(QM) energies were set to zero. Similarly, if the QM calculations terminated due to excessive steric repulsion, the MIF(QM) energies at the corresponding grid points were also set to zero. Zimmermann et al. previously applied similar calculations to analyze halogen bonds,^34^ while Kiani et al. proposed intermolecular interaction potential maps for visualizing interactions on an isodensity surface of a target molecule.^35^ However, the method used in the present study differs from these approaches as it is specifically optimized for 3D analyses of weak hydrogen bonds, as detailed in our prior works.^2,3^

### Contractions of the MIFs(QM)

The obtained MIFs(QM) for weak hydrogen bonds are intended for application in analyzing weak hydrogen bonds formed in protein/ligand systems. For these analyses, converting the grid data of MIFs(QM) into a more practical format is necessary. In this study, we contracted the calculated MIFs(QM) using Gaussian expansions. The contraction is expected to be useful not only for analyzing protein/ligand structures but also for constructing databases or libraries and developing scoring functions^36^ for ligand docking calculations in future works. The approximation functions of the calculated MIFs(QM) are represented as a linear combination of Gaussian functions as follows:^3^
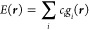
1where *c*_*i*_, *g*_*i*_, and ***r*** represent the expansion coefficient, Cartesian Gaussian function (CGF), and position vector, respectively. CGFs are defined as follows:
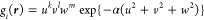
2*u*, *v*, and *w* are the components of the vector **r**. *k*, *l*, and *m* are integers that characterize the order of the CGF. α represents the exponents of the CGFs, which are predefined. The expansion coefficients *c*_*i*_ are determined by minimizing the function Δ (eq 3):
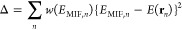
3*n*, *E*_MIF,*n*_, and ***r***_*n*_ represent the index of a grid point, the MIF(QM) energy at the grid point *n*, and the position vector of the grid point *n*, respectively. *w*(*E*_MIF,*n*_) is a weighting function (WF), which was not used in our previous work^3^ but is newly introduced in this study to improve the reproducibility of MIF(QM) values in the regions with high MIF(QM) values. The weighing function is defined as follows:
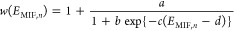
4

This is a sigmoid function with *a*, *b*, *c*, and *d* parameters. The coefficients *c*_*i*_ that minimize Δ are obtained through matrix calculations using eq 5:
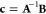
5

The components of the matrices **A** and **B** (*A*_*ji*_, *B*_*j*_) are defined as follows:
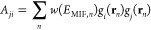
6a
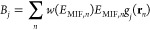
6b

The exponents α for respective CGFs are determined using the following eq 7.
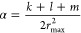
7*r*_max_ is the distance from the origin to the maximum point of the CGF. The optimum value of *r*_max_ is estimated to align closely with the optimal interaction distances of weak hydrogen bonds. For example, the distance for CH/π interactions is defined as the distance from the center of the aromatic ring of an acceptor to the carbon atom of a donor CH group. In this study, five functions with different α values were adopted for each type of CGF. Consequently, the following CGFs were included: 5 s-, 15 p-, 30 d-, 50 f-, 75 g-, 105 h-, 140 i-, and 180 j-type CGFs, totaling 600 CGFs. The MIFs calculated using the approximation functions *E*(*r*) are denoted as MIF(func, CH_4_) and MIF(func, BZ), respectively, according to the probe molecule.

### CSD Analyses and Density(CSD) Maps

Short contacts between molecules in crystal structures registered in the CSD were analyzed using the CONQUEST program.^32^ The analyses required definitions of a target chemical group (central group) and a contact group interacting with the target. Pyridine and pyrimidine substructures were defined as central groups, while X–CH_3_, X=CH_2_, C_5_X_5_CH (phenyl ring CH), and X≡CH (X: any atom) were defined as contact groups. The search range for contact was set to 7 Å. Ions, polymers, and organometallic compounds were excluded in the analyses with the detailed conditions listed in Table S2. Scatter plots were generated from the output files obtained by CONQUEST using the IsoGen program.^33^ To manage cases where the number of contact group fragments was excessively high, fragments were randomly selected by the IsoGen program to reduce their number. The selection process was automated by the program and could not be manually adjusted. Density(CSD) maps were then generated from the scatter plots using the Isostar program.^31,33^ Additionally, previously published density(CSD) maps for quinoline and 1,3,5-triazine substructures were reanalyzed.^31^ Isostar format files were downloaded from the Isostar library (https://isostar.ccdc.cam.ac.uk/html/isostar.html) and density(CSD) maps with methyl CH and aromatic CH as contact groups were depicted using the Isostar program. CSD analyses depend on the amount of available data in the database. Therefore, density(CSD) maps were calculated for pyridine, pyrimidine, 1,3,5-triazine, and quinoline substructures, which have sufficient crystalline data in the CSD. In this study, the density(CSD) maps are denoted as density(CSD, X-CH_3_) according to the contact group.

In statistical analyses based on the CSD, it is generally explicitly or implicitly assumed that the frequency of interaction geometries observed in crystal structures reflects the stability of the interactions. However, the validity of this assumption depends on the type of interactions.^37^ The most practical strategy to validate this assumption is to compare the results of the CSD analyses with the interaction energies calculated by QM. In the present study, we compared the density(CSD) and MIF(QM) maps to validate this assumption.

### Density(PDB) Maps

The crystal data of the protein/ligand complexes registered in the PDB provide valuable insights for investigating intermolecular interactions, although their resolutions are lower than those in the CSD. Thus, we calculated the density maps using the crystal data of the protein/ligand complexes in PDB. These density maps are denoted as density(PDB) maps. Pyridine, pyrimidine, 1,3,5-triazine, quinoline, and indazole substructures were used as central groups. The alkyl CH groups in the Ala, Ile, Leu, Pro, and Val side chains were defined as contact groups. The obtained density(PDB) maps were denoted as density(PDB, alkyl CH). The calculation procedure for density(PDB) maps was as follows: (1) PDB format files were downloaded from the PDB web site (https://www.rcsb.org), (2) protein/ligand complex structures were aligned, (3) scatter plots were generated, and (4) density(PDB) maps were generated. Protein–ligand complex structures containing ligands with the target substructure were searched using an advanced search function on the PDB Web site. The substructures were specified in the SMILES format. In the case of pyridine and pyrimidine substructures, we used the complex structures determined by X-ray diffraction with a resolution within 2 Å for the analyses. In the case of 1,3,5-triazine, quinoline, and indazole substructures, the upper limit of the resolutions was set at 3 Å, because the numbers of hit structures were insufficient when the limit was set at 2 Å. For the 1,3,5-triazine and indazole substructures, structures that met the above conditions were used for the analyses (227 and 523 structures, respectively). In the case of the pyridine, pyrimidine, and quinoline substructure, more than 1,000 complex structures met the above conditions. Thus, we randomly selected 500 structures per target for analysis. The random selection was performed based on computationally generated random numbers. The protein–ligand complex structures were aligned such that the substructures within the ligands were superimposed on the reference structure. The model structures used in MIF(QM) calculations were adopted as reference structures. Alignments of the protein/ligand structures were performed using Schrödinger Maestro (Schrödinger, LLC, New York, NY, 2017). A list of PDB IDs of the structures used in the analyses is provided in Supporting Information. The scatter plots and density(PDB) maps were obtained using our in-house code. The positions of the fragments were translated and copied considering the symmetry of the target substructures before the density calculations. For generating the density(PDB) maps, the grid size was set to 0.5 Å, and the densities were smoothed by averaging the values of each grid point with those of its 26 adjacent grid points.

### Computational Details

All DFT calculations were performed using the Gaussian16 program.^38^ The M06-2X functional^39^ was used in combination with the aug-cc-pVDZ basis set. The basis set superposition error (BSSE) was corrected using the counterpoise (CP) method.^40^ Previous benchmark calculations indicate that the M06-2X/aug-cc-pVDZ(CP) method accurately reproduces interaction energies of CH/π interactions obtained by CCSD(T) with a complete basis set, with an error margin of approximately 0.5 kcal/mol.^41^ Therefore, this calculation method was adopted in the present study. For the double-ring/benzene system, M06-2X/6-31G(d,p) calculations were used, as the number of basis sets became too large when using the aug-cc-pVDZ basis set. The validity of the 6-31G(d,p) basis set was confirmed by comparing the MIFs(QM, BZ) maps of pyridine, calculated using the aug-cc-pVDZ and 6-31G(d,p) basis sets (Figure S1). The arrangement and orientation of probe molecules for MIF(QM) calculations, as well as the fitting calculations to contract MIFs(QM), were performed using an in-house code. Matrix calculations were conducted using BLAS-LAPACK (https://www.netlib.org/lapack/). Molecular models were visualized using the Visual Molecular Dynamics (VMD) program.^42^

## Results and Discussion

### Comparison between the MIF(QM) and Density(CSD) Maps

**Figure 3:**
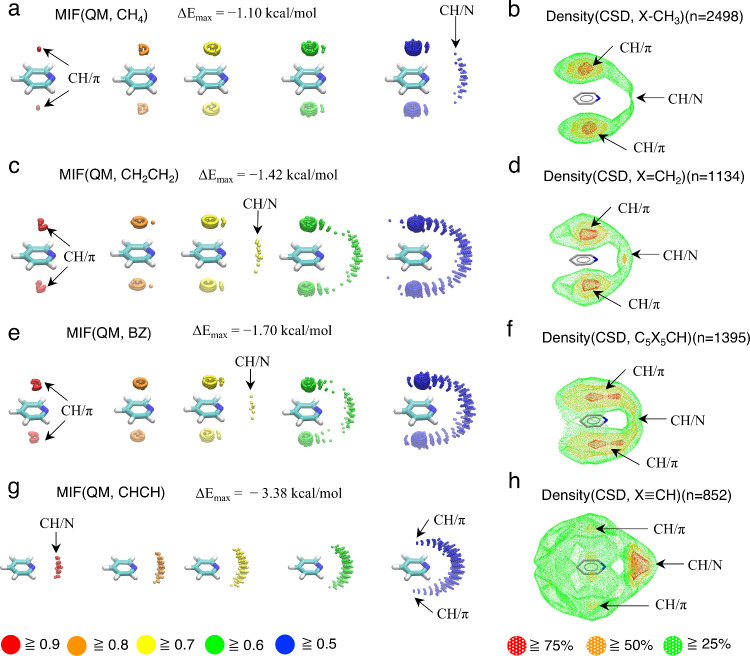
MIF(QM) maps of pyridine with (a) CH_4_, (c) CH_2_CH_2_, (e) benzene, and (g) CHCH probes. 3D alternations of MIFs(QM) are depicted by spheres colored according to the MIF energy. MIFs(QM) were obtained by M06-2X/aug-cc-pVDZ(CP) calculations. Density(CSD) maps with a pyridine substructure as the central group and (b) X–CH_3_, (d) X=CH_2_, (f) C_5_X_5_CH, and (h) X≡CH as contact groups. The areas with densities of 75%, 50%, and 25% or higher of the maximum density are shown in red, orange, and green, respectively. The densities were evaluated based on the positions of (b, h) carbon atoms or (d, f) hydrogen atoms of donor CH groups. All density(CSD) maps were obtained by CSD analyses using IsoGen and Isostar programs.

Nitrogen-containing heterocyclic compounds are predicted to form CH/π and CH/N interactions; however, this assumption has not been validated, and their 3D features remain unexplored. To address this, we investigated the 3D characteristics of CH/π and CH/N interactions around model molecules using MIF(QM) and CSD analyses. The calculated MIF(QM) map with a CH_4_ probe (MIF(QM, CH_4_)) for the pyridine model is shown in Figure 3a. Regions with MIF(QM) energy values of 0.9 or higher, representing the most favorable interaction areas, are located above and below the aromatic ring of pyridine (Figure 3a, marked by arrows), corresponding to CH/π interaction regions. The interaction energy of the most stable point (Δ*E*_max_) is −1.10 kcal/mol, indicating that a normalized MIF(QM) energy of 1.0 corresponds to an interaction energy of −1.10 kcal/mol. Regions with MIF(QM) energy values of 0.5 or higher are observed around the nitrogen atom of pyridine (marked by an arrow), suggesting CH/N interaction regions, albeit weaker than the CH/π interactions. These results suggest that CH/π interactions are more favorable than CH/N interactions in pyridine/C–H(sp^3^) systems. Figure 3b shows a density(CSD) map with X–CH_3_ contact groups and pyridine substructures as the central group. Areas with densities of 75% or higher of the maximum value are found above and below the aromatic ring of pyridine (marked by arrows), corresponding to CH/π interactions formable areas. Additionally, regions with densities of 25% or higher can be observed around the nitrogen atom, consistent with CH/N interaction formable area (marked by arrow). As statistical mechanics suggest that energetically stable states are more frequently observed, the density(CSD) map derived from CSD analysis aligns well with the calculated MIF(QM) map. The calculated MIF(QM, CH_2_CH_2_) for pyridine is shown in Figure 3c. CH/π interaction formable areas above and below the aromatic ring are evident (marked by arrows), with an Δ*E*_max_ of −1.42 kcal/mol, indicating stronger CH/π interactions in the pyridine/CH_2_CH_2_ system compared to pyridine/CH_4_. Prior QM studies have shown that CH/π interaction strength depends on the acidity of the donor group,^43−45^ with higher acidity correlating to stronger interactions. The MIF(QM) results align with this trend. The feature of the MIF(QM, CH_2_CH_2_) is similar to that of MIF(QM, CH_4_). However, the CH/N interaction strength relative to CH/π interactions is higher in MIF(QM, CH_2_CH_2_) than in MIF(QM, CH_4_), as shown by MIF energy values of 0.7 (Figure 3c, marked by arrow) and 0.5 (Figure 3a, marked by arrow), respectively. The density(CSD, X=CH_2_) map (Figure 3d), shows regions with densities of 75% or higher above and below the aromatic ring (marked by arrows) and regions with densities of 50% or higher around the nitrogen atom of the pyridine scaffold (marked by arrow). These results suggest stronger relative CH/N interaction strengths in pyridine substructure/CH(sp^2^) systems compared to pyridine substructure/CH(sp^3^) systems, consistent with the MIF(QM) calculations. Previous QM studies proposed that the strengths of CH/π and CH/N interactions depend on the acidity of the donor group.^43−47^ The present study, utilizing MIF(QM) calculations and CSD analyses, confirms the validity of this insight. Furthermore, this work is the first to propose a change in the relative strength between CH/π and CH/N interactions. The MIF(QM, BZ) map is similar to the MIF(QM, CH_2_CH_2_) map due to the sp^2^ nature of the benzene carbons (Figure 3e). Comparing the density(CSD, C_5_X_5_CH) map with the density(CSD, X–CH_3_) map suggests that the relative strengths of CH/N interactions in pyridine substructure/C_5_X_5_CH systems are stronger than those in pyridine substructure/X–CH_3_ systems (Figure 3f). This trend is also consistent with the MIF(QM) calculation results. The MIF(QM, CHCH) map indicates an inversion in the relative strengths of CH/π and CH/N interactions (Figure 3g, marked by arrows). Specifically, CH/N interactions around the nitrogen atom are more stable than CH/π interactions above and below the aromatic ring (marked by arrows). This tendency is also evident in the corresponding density(CSD, X≡CH) map (Figure 3h). Additionally, the Δ*E*_max_ of MIF(QM, CHCH) is –3.38 kcal/mol, making it the strongest among the calculated MIFs(QM) maps for pyridine.

**Figure 4:**
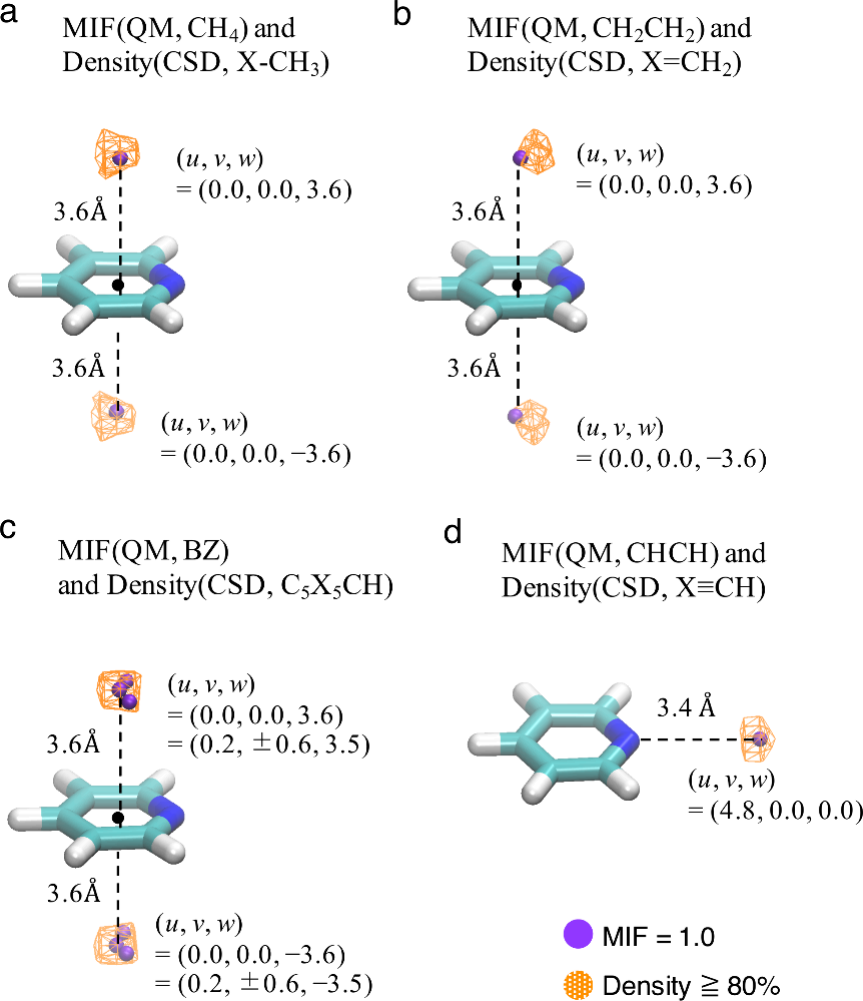
Superimposition of the (a) MIF(QM, CH_4_) map of pyridine and density(CSD, X–CH_3_) map of the pyridine substructure, (b) MIF(QM, CH_2_CH_2_) map of pyridine and density(CSD, X=CH_2_) map of the pyridine substructure, (c) MIF(QM, BZ) map of pyridine and density(CSD, C_5_X_5_CH) map of the pyridine substructure, and (d) MIF(QM, CHCH) map of pyridine and density(CSD, X≡CH) map of the pyridine substructure. The positions of the grid points with the most stable MIF(QM) energies are indicated by purple spheres. Areas with densities of 80% or higher of the maximum value are shown as orange meshes. In all density(CSD) maps, the densities are calculated based on the positions of carbon atoms, as the grid points for MIF(QM) calculations correspond to the positions of the carbon atoms in the probe molecules. The MIFs(QM) were obtained by M06-2X/aug-cc-pVDZ(CP) calculations, and the density(CSD) maps were obtained by CDS analyses using the IsoGen program and our in-house code.

The MIF(QM) and density(CSD) maps were superimposed using their respective donor groups for comparison (Figure 4). Figure 4 suggests that the positions of grid points with the most stable MIF(QM) energies align with areas having densities of 80% or higher of the maximum value in the density(CSD) maps. For CH/π interactions, the distances between the grid points with the most stable MIF(QM) energies and the centers of aromatic rings are 3.6 Å (Figures 4a–c). Given that the typical CH bond length is about 1.0 Å, the distances between the donor hydrogens of the probe molecules and the ring centers are about 2.6 Å. This value is consistent with previously reported average distances for CH/π interactions.^19^ For CH/N interactions, the distance between the grid point with the most stable MIF(QM) energy and the nitrogen atom is 3.4 Å (Figure 4d). This suggests a predicted distance of about 2.4 Å between the donor hydrogen of the probe molecule and the acceptor nitrogen atom, aligning with previously reported values.^20,46^ Notably, the density(CSD) maps in Figure 4 are additionally calculated using our in-house code, as the MIF maps cannot be converted into a format readable by the Isostar program. In these additional calculations, densities were derived from scatter files (.mol2 format) generated by the IsoGen program and outputted in VMD format (.dx format). The grid size was set to 0.4 Å, and the densities were smoothed by averaging the values of each grid point with those of its 26 adjacent grid points.

The calculated MIF(QM, CH_4_) map of pyrimidine is shown in Figure S2a. Similar to pyridine, CH/π interaction formable areas can be observed above and below the aromatic ring. Pyrimidine contains two nitrogen atoms, each capable of forming a CH/N interaction. Consequently, CH/N interaction formable areas are located around the nitrogen atoms. These interaction formable areas are also reflected in the density(CSD, X–CH_3_) map (Figure S2b). The MIF(QM) maps shown in Figure S2a,c,e,g suggest that the overall strength of interactions between pyrimidine and a CH group increases with the acidity of the donor group (Δ*E*_max_ values range from −0.93 to −2.89 kcal/mol). Similar to pyridine, the relative stability of CH/N interactions compared to CH/π interactions also increases (Figure S2a,c,e,g). In the CSD analyses of pyrimidine substructures, the available crystal data include fewer than 300 samples for X=CH_2_ and X≡CH contact groups. Although this sample number is relatively low, the density(CSD) maps still provide valuable insights and support the trends observed in the MIF(QM) calculations (Figure S2b,d,f,h).

The density (CSD) maps depend on the selected atom within the contact groups used to calculate density. To assess this dependence, the density(CSD) maps of pyridine and pyrimidine have been recalculated with different atom selections, as shown in Figures S3 and S4 in the Supporting Information. Although slight differences can be observed based on the selected atoms, the general trends noted in Figures 3 and S2 have been reproduced across all the density(CSD) maps. Notably, Figure 3 shows the typical maps selected to facilitate reader understanding while avoiding redundancy.

**Figure 5:**
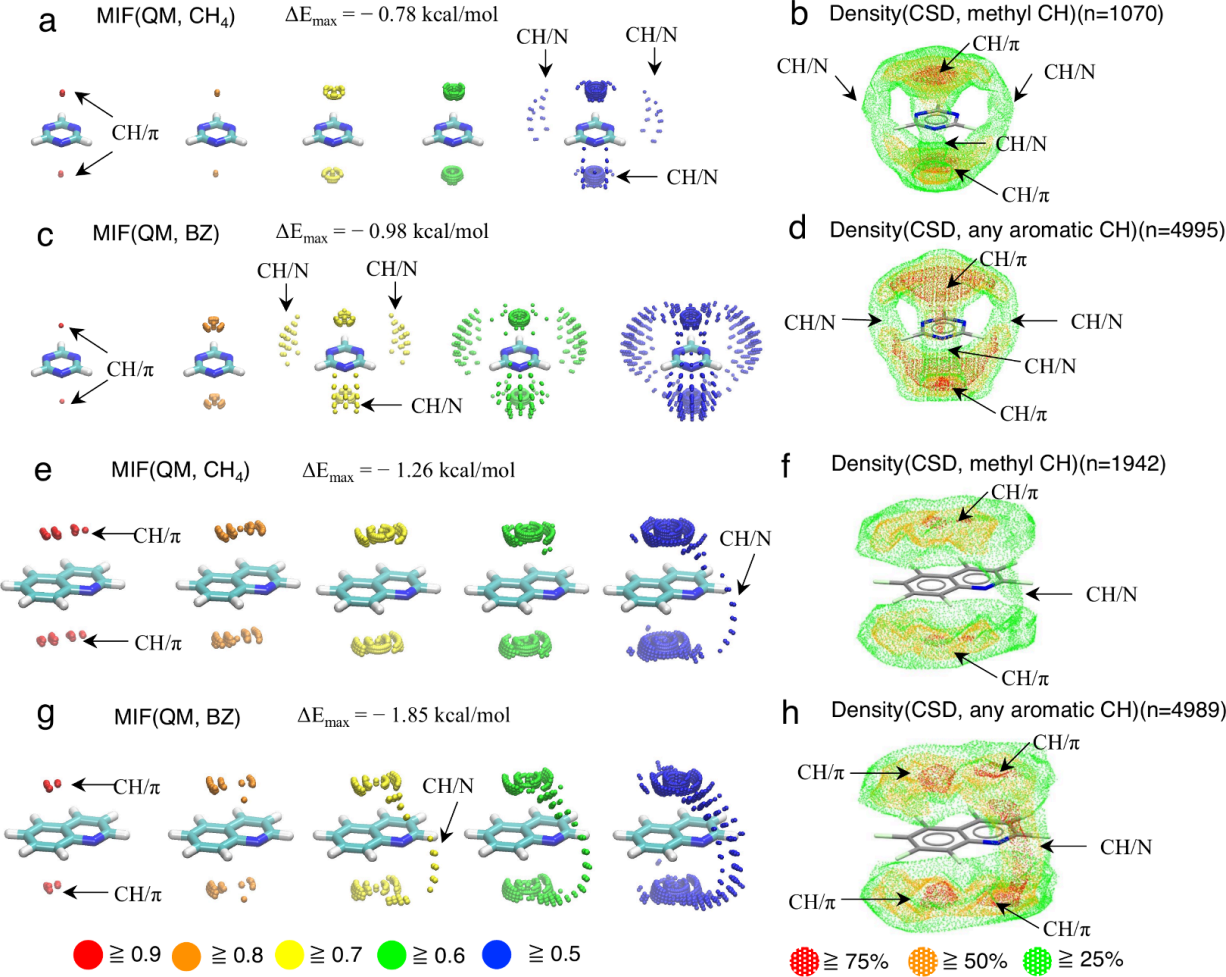
MIF maps of 1,3,5-triazine with (a) CH_4_ and (c) benzene probes. MIF(QM) maps of quinoline with (e) CH_4_ and (g) benzene probes. 3D alternations of MIFs(QM) are depicted by spheres colored according to the MIF(QM) energy. The MIFs(QM) were obtained by M06-2X/aug-cc-pVDZ(CP) calculations, except for MIF(QM, BZ) of quinoline, where M06-2X/6-31G(d,p) calculations were used. Density(CSD) maps of 1,3,5-triazine substructure with (b) methyl and (d) any aromatic CH contact groups. Density(CSD) maps of quinoline substructure with (f) methyl and (h) any aromatic CH contact groups. The areas with densities of 75%, 50%, and 25% or higher of the maximum value are shown in red, orange, and green, respectively. These densities are calculated based on the positions of hydrogen atoms. All density(CSD) maps were obtained by CSD analyses using IsoGen and Isostar programs.

The density(CSD) maps of 1,3,5-triazine and quinoline substructures published in the Isostar library were reanalyzed for comparison with the calculated MIFs(QM) (https://isostar.ccdc.cam.ac.uk/html/isostar.html).^31^ Density(CSD) maps with methyl CH and aromatic CH groups were analyzed. Unfortunately, data for density(CSD) maps with X=CH_2_ and X≡CH groups were unavailable. Figure 5 shows the MIF(QM, CH_4_) and MIF(QM, BZ) maps alongside the density(CSD) maps. CH/π interaction formable areas are evident above and below the 1,3,5-triazine plane (Figure 5a–d). CH/N interaction formable areas are located around each of the three nitrogen atoms (Figure 5a–d). The relative strength of CH/N interactions compared to CH/π interactions is greater in the 1,3,5-triazine/benzene system than in the 1,3,5-triazine/CH_4_ system. The trend is consistent across pyridine, pyrimidine, and 1,3,5-triazine. For MIF(QM, BZ) calculations involving quinoline and other double-ring model molecules, the M06-2X/6-31G(d,p) calculation was used. The CH/π interaction formable areas above and below the quinoline plane are wider than those observed for single-ring molecules (Figures 5e–h). The CH/N interaction formable area is evident around the nitrogen atom (Figure 5e–h). Additionally, the relative strength of CH/N interactions compared to CH/π interactions is greater in the quinoline/benzene system than in the quinoline/CH_4_ system (Figure 5e–h).

In the protein/ligand systems, the donor CH groups involved in CH/π or CH/N interactions are typically alkyl or aromatic when the acceptor is a nitrogen-containing heterocyclic substructure of a ligand. Therefore, we focused on the MIF(QM) maps with CH_4_ and benzene probes for the remaining 14 model molecules. The MIF(QM, CH_4_) and MIF(QM, BZ) maps of these models are shown in Figures S5–S12. These MIF(QM) maps exhibit trends consistent with those discussed previously. However, for pyridazine, 1,2,4-triazine, and purine, which possess two nitrogen atoms with lone pairs in close proximity, CH/N interactions are stronger than CH/π interactions when the probe molecule is benzene. The average interaction energies for CH/π interactions are predicted to be −1.1 and −1.8 kcal/mol for CH_4_ and benzene probes, respectively. These average interaction energies are slightly weaker than those of benzene/CH_4_ (−1.3 kcal/mol) and benzene/benzene (−2.3 kcal/mol) systems, respectively (Figure S8c,d). Previous *ab initio* studies reported CH/π interaction energies of −1.45 and −2.46 kcal/mol for benzene/methane and benzene/benzene systems, respectively.^43,48^ The average interaction energies for CH/N interactions are predicted to be −0.6 and −1.3 kcal/mol for CH_4_ and benzene probes, respectively. These values align with those reported in previous QM calculations using simple model molecules.^46^ No CH/N interaction formable areas are evident around pyrrole and indole molecules, as these structures lack an acceptor nitrogen atom.

As discussed above, the MIFs(QM) and density(CSD) maps of the aromatic nitrogen-containing heterocyclic compounds provide the following three insights.

1) The 3D feature of the CH/π interaction area above and below the aromatic ring.
2) The 3D feature of the CH/N interaction area around the nitrogen atoms.
3) The relative stability of CH/N interactions compared to CH/π interactions increases as the acidity of the donor CH group rises.

Additionally, we again confirmed that the strength of CH/π and CH/N interactions increases with the acidity of the donor CH group.^43−47^ In protein/ligand systems, CH/π interactions are generally predicted to be stronger than CH/N interactions when the donor groups are CHs from methyl or phenyl groups. However, in the case of pyridazine, 1,2,4-triazine, and purine, CH/N interactions with benzene substructures are predicted to be stronger than CH/π interactions.

We confirmed that the calculated MIFs(QM) are consistent with the density(CSD) maps. Therefore, we conclude that the MIF(QM) calculation method provides reliable 3D information on CH/π and CH/N interactions. Additionally, this suggests that the frequencies of the interaction geometries observed in the crystal structures in the CSD qualitatively correlate with the interaction energies calculated by QM in these systems. Moreover, as the MIF(QM) calculations do not rely on experimental data, they serve as a powerful tool for investigating the 3D features of weak hydrogen bonds in the absence of experimental data availability.

### Contraction of Calculated MIFs and Its Application to Protein/Ligand Interaction Analyses

**Figure 6:**
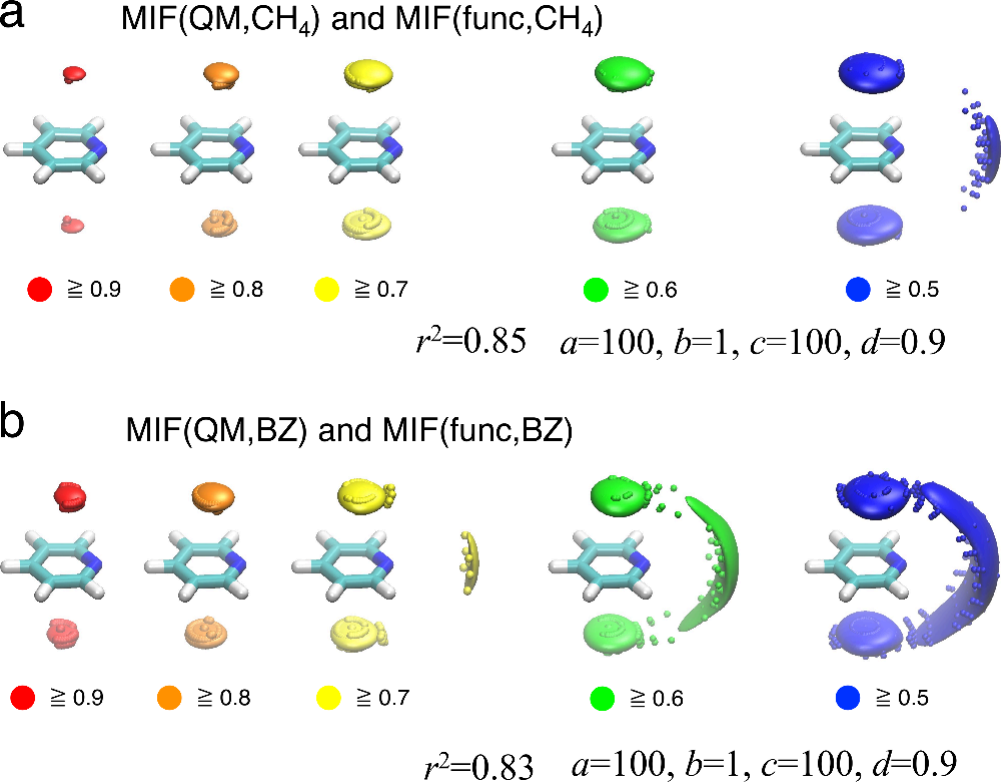
Results of contractions of MIFs(QM) maps: Superposition of the (a) MIF(QM, CH_4_) and MIF(func, CH_4_) and (b) MIF(QM, BZ) and MIF(func, BZ) maps for pyridine. 3D variations in the MIFs(QM) maps are depicted by spheres colored according to the MIF(QM) energy. The MIFs(func) maps are represented as surfaces with the same color scheme.

Figure 6 shows the MIF(func, CH_4_) and MIF(func, BZ) maps of the pyridine molecule calculated using the approximation functions *E*(**r**) derived from the contraction of MIF(QM, CH_4_) and MIF(QM, BZ), respectively. The MIFs(func) maps were overlaid on the corresponding MIFs(QM). For the calculation of MIFs(func), a total of 4,173,281 grid points (161 × 161 × 161) with an interval of 0.1 Å were defined. Figure 6a,b show that the MIF(func, CH_4_) and MIF(func, BZ) maps successfully reproduce the shapes and normalized energy values of MIF(QM, CH_4_) and MIF(QM, BZ), respectively. The correlation between MIF(QM, CH_4_) and MIF(func, CH_4_) is shown in Figure S13a, with a coefficient of determination (*r*^2^) of 0.85. Similarly, the correlation between MIF(QM, BZ) and MIF(func, BZ) is shown in Figure S13b, with an *r*^2^ value of 0.83. Conventional potential functions for MIF calculations, such as those for hydrogen bonding and lipophilicity, have been widely applied in molecular modeling.^49−58^ Additionally, knowledge-based methods like Superstar and Isostar are well-established tools in molecular modeling.^31,59−62^ As a QM-level expansion of these methodologies, the approximation functions proposed in this study can be applied to various modeling efforts, particularly those involving weak hydrogen bonds such as CH/π and CH/N interactions. The features of the MIFs(func, CH_4_) and MIFs(func, BZ) maps for the 15 chosen compounds are shown in Figures S5–S12, and the optimized parameters are given in Supporting Information.

**Figure 7:**
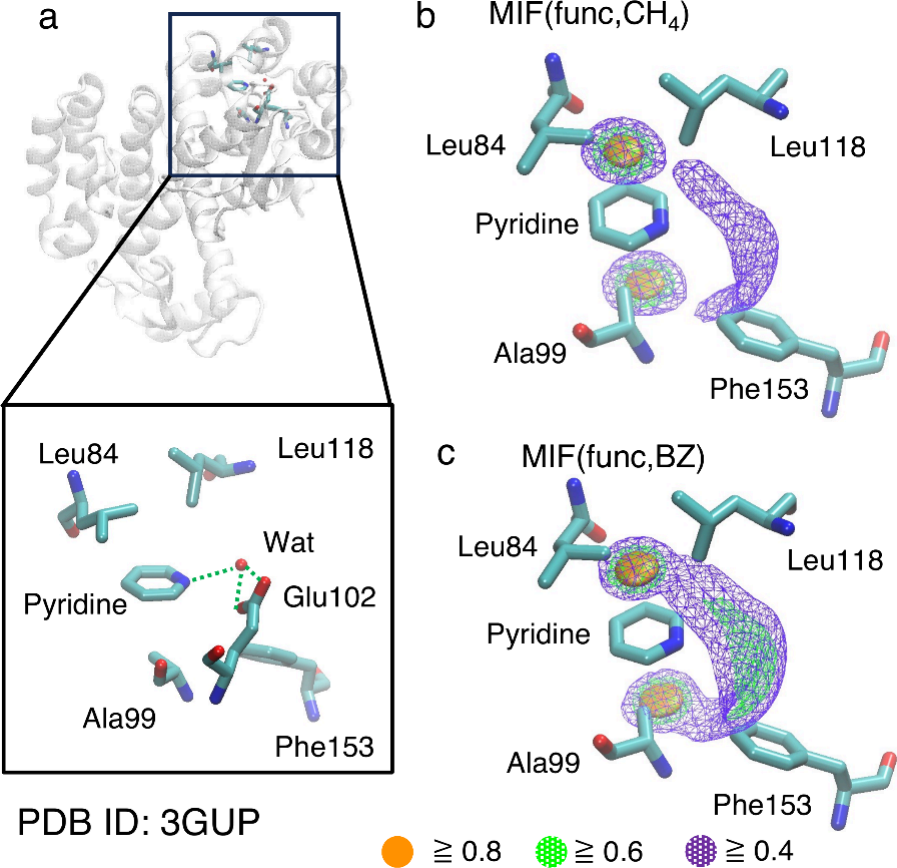
(a) T4 lysozyme M102E/L99A mutant/pyridine complex structure (PDB ID: 3GUP). (b) MIF(func, CH_4_) and (c) MIF(func, BZ) maps calculated around the pyridine molecule binding to the mutant T4 lysozyme. The pyridine molecule and amino acid residues are depicted as stick models. The interaction formable areas with MIF(func) energy values of 0.8 or higher are represented as orange surfaces. The interaction formable areas with MIF(func) energy values of 0.6 and 0.4 or higher are depicted as green and purple meshes, respectively.

As a proof-of-concept calculation for protein/ligand complex analysis using MIFs(func), we analyze the T4 lysozyme M102E/L99A mutant/pyridine complex (PDB ID: 3GUP). In the complex structure, a water-mediated hydrogen bond of Pyridine-Water-Glu102 is formed (Figure 7a).^63^ This water-mediated hydrogen bond is predicted to be a key factor in the formation of the mutant T4 lysozyme/pyridine complex. In addition to the hydrogen bond, the pyridine molecules have the potential to form CH/π and CH/N interactions. To evaluate the possibilities of these interactions, we calculated MIF(func, CH_4_) and MIF(func, BZ) around the pyridine molecule in the complex structure. A total of 8,000,000 grid points (200 × 200 × 200) with an interval of 0.1 Å were defined around the ligand pyridine molecule. The molecular coordinate system (*o-uvw*) was defined on the pyridine molecule within the complex structure, and a rotational matrix that transforms laboratory coordinates (*o-xyz*) to molecular coordinates (*o-uvw*) was obtained. Using this matrix, the *x*, *y*, and *z* components of each grid point in laboratory coordinates were transformed into molecular coordinates (*u*, *v*, *w*). The MIF(func) value at each grid point was then calculated by adding the *u*, *v*, and *w* values to the function *E*(*r*). This procedure follows our previously reported method.^3^Figure 7b shows that the aliphatic side chains of Leu84, Ala99, and Leu118 are located near the CH/π interaction formable areas above and below the pyridine ring plane. While the carbon atoms of these side chains are slightly shifted from the favorable CH/π interaction formable areas (≥0.8), the shift distances are shorter than CH bond lengths. Considering the resolutions of the X-ray crystal structure and thermal fluctuation of the side chains, the formation of the CH/π interaction between the pyridine molecule and these side chains is plausible. Figure 7c demonstrates MIF(func, BZ) also predicts CH/π interaction formable areas above and below the pyridine ring plane. However, no atomic group containing benzene substructure is present near these areas, indicating that no CH/π interaction involving an aromatic group is formed. Figure 7c shows that the benzene ring of Phe153 residue is positioned close to the CH/N interaction formable area around the nitrogen atom of pyridine. This suggests the potential formation of an additional CH/N interaction between pyridine and the Phe153 residue. Although the CH/π and CH/N interactions predicted by MIFs(func) are expected to be weak, the results indicate that the pyridine binding site in the T4 lysozyme mutant has a 3D structure optimized for pyridine binding. Furthermore, MIFs(func) suggest that CH/π and CH/N interactions may influence the binding orientation of the ligand, as the water-mediated hydrogen bond alone cannot fully determine the ligand’s orientation.

**Figure 8:**
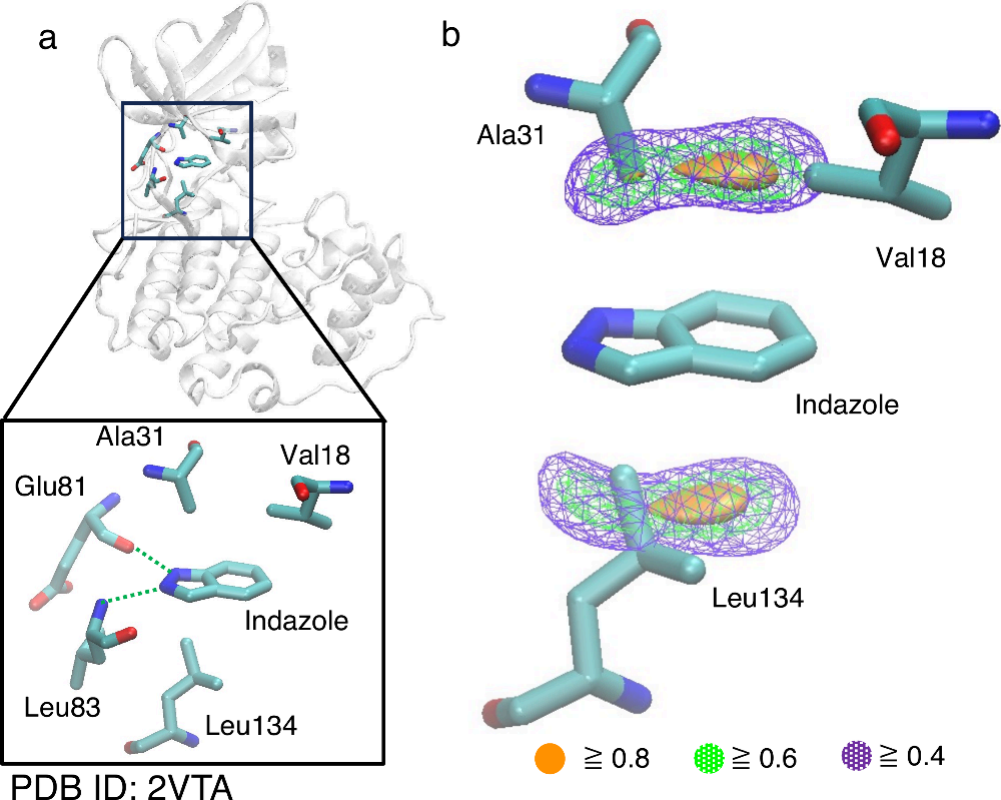
(a) Cyclin-dependent kinase 2 (CDK2)/indazole complex structure (PDB ID: 2VTA). (b) MIF(func, CH_4_) map calculated around the indazole molecule bound to CDK2. The indazole molecule and amino acid residues are depicted as stick models. The interaction formable areas with MIF(func) energy values of 0.8 or higher are represented as orange surfaces. The interaction formable areas with MIF(func) energy values of 0.6 and 0.4 or higher are depicted as green and purple meshes, respectively.

As another example, MIF(func, CH_4_) was calculated around the indazole molecule bound to cyclin-dependent kinase 2 (CDK2) (Figure 8). The CDK2/indazole complex structure (PDB ID: 2VTA) suggests the formation of two hydrogen bonds: one between the carbonyl oxygen of Glu81 and the N–H group of the indazole molecule and another between the N–H group of the Leu83 and the nitrogen atom of the indazole molecule (Figure 8a).^64^ These hydrogen bonds are predicted to be key factors contributing to the complex formation. Within the ligand binding site of CDK2, alkyl groups of Val18, Ala31, and Leu134 are positioned above and below the indazole ring. The CH/π interaction formable areas, widely distributed above and below the indazole ring, cover these alkyl groups (Figure 8b). This observation suggests the formation of CH/π interactions between CDK2 and the indazole molecule. Two-ring nitrogen-containing heterocycles like indazole exhibit broad CH/π interaction formable areas, making them favorable for forming CH/π interactions.

We demonstrated two examples of applying MIF(func) calculations to analyze CH/π and CH/N interactions in protein/ligand complexes. These examples underscore that protein/ligand interaction analyses using MIFs(func) provide valuable insights into molecular recognition by proteins.

### Density(PDB) Maps

**Figure 9:**
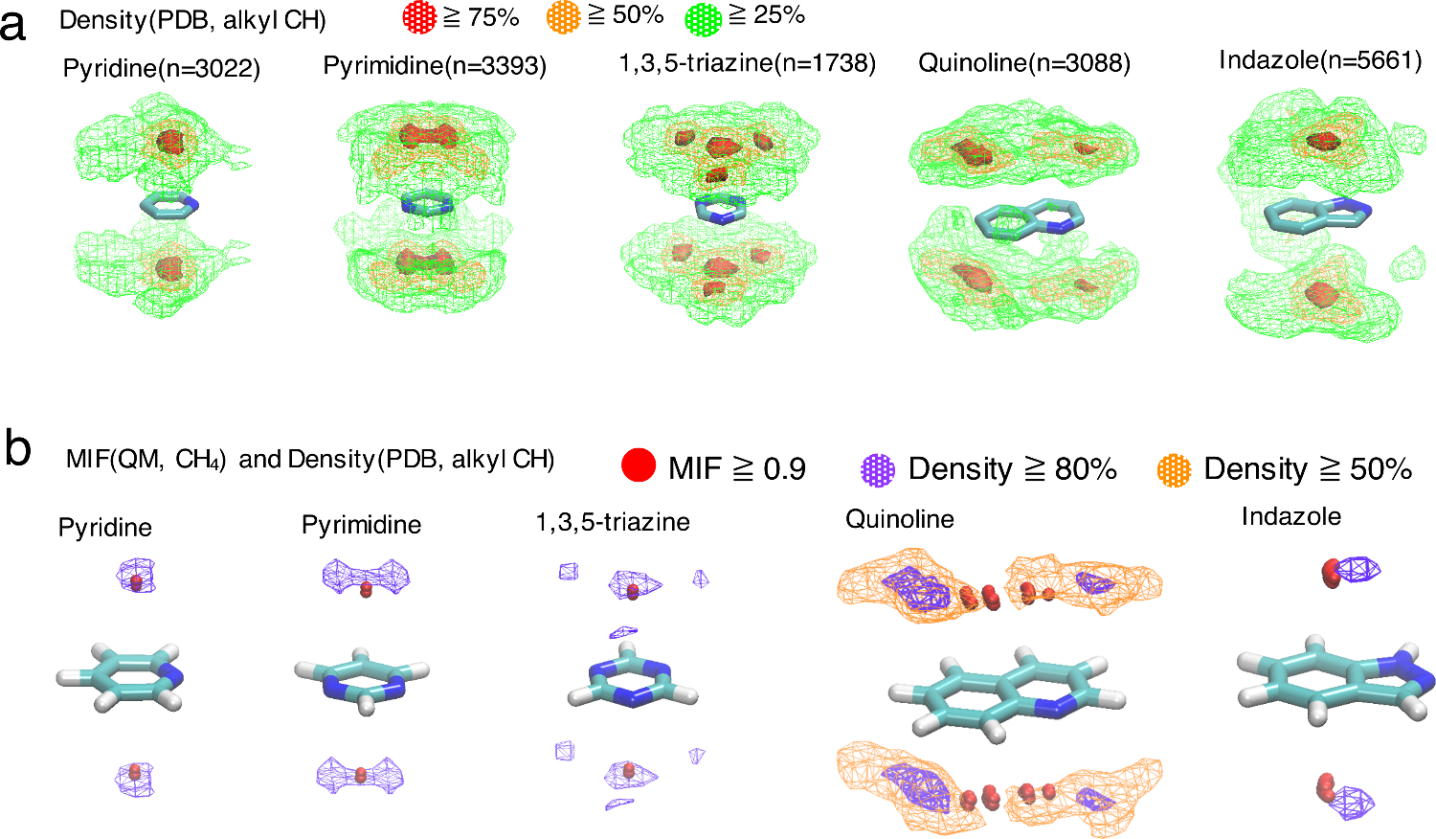
(a) Density(PDB, alkyl CH) maps of the pyridine, pyrimidine, 1,3,5-triazine, quinoline, and indazole substructures. The areas with densities of 75%, 50%, and 25% of the maximum value or higher are shown in red, orange, and green, respectively. The densities were evaluated based on the positions of carbon atoms of donor CH groups. All density(PDB) maps were obtained based on the analyses of protein/ligand complexes in PDB using in-house code. (b) Superimpositions of the MIF(QM, CH_4_) and density(PDB, alkyl CH) maps of pyridine, pyrimidine, 1,3,5-triazine, quinoline, and indazole. Areas with densities of 80% and 50% (only quinoline) of the maximum value or higher are shown as purple and orange meshes, respectively. Areas with MIF(QM) energy of 0.9 or higher are shown in red spheres. MIFs(QM) were obtained by M06-2X/aug-cc-pVDZ(CP) calculations.

In the above section, we showed examples of the applications of MIFs(func) to protein/ligand complex analyses. The 3D features of the CH/π and CH/N interactions formed in protein/ligand systems were of interest. Herein, we calculate the density(PDB) maps and compare them with those of MIFs(QM). Figure 9a shows the density(PDB, alkyl CH) maps of the pyridine, pyrimidine, 1,3,5-triazine, quinoline, and indazole substructures.

The density(PDB, alkyl CH) maps of pyridine, pyrimidine, 1,3,5-triazine, and quinoline were generally in good agreement with the density(CSD, X−CH_3_) maps shown in Figures 3, 5, and S2. CH/π interaction areas are formed above and below the aromatic rings for respective substructures. The density(PDB, alkyl CH) map of quinoline showed four maximum points above and below the aromatic ring. The maximum points were closer to the edge of the quinoline ring than those observed in the density(CSD, X–CH_3_) map. The shifts in the maximum points may be due to differences in the environments between the crystal structures of small molecules and protein/ligand complexes. Figure 9b shows a comparison of the density(PDB, alkyl CH) and MIF(QM, CH_4_) maps. For pyridine, pyrimidine, 1,3,5-triazine, and indazole, the areas with 80% densities(PDB, alkyl CH) or higher than the maximum values were completely aligned with the areas with MIF(QM) energy of 0.9 or higher. In the case of quinoline, the maximum points of density(PDB, alkyl CH) slightly shifted from the area with an MIF(QM) energy of 0.9 or higher. The differences were from 0.5 to 1.0 Å, which are efficiently small considering the resolutions of X-ray crystal structures. Thus, we concluded that the MIF(QM, CH_4_) maps aligned well with the density(PDB, alkyl CH) maps of the systems. That is, the frequencies of the interaction geometries observed in the protein/ligand complex structures in the PDB were qualitatively correlated with the interaction energy calculated by QM in these systems. This suggests the validity of the MIF(QM) calculations in these systems.

## Conclusions

We demonstrated that combining MIF(QM) calculations with CSD analysis enables the characterization of the 3D features of CH/π and CH/N interactions around nitrogen-containing heterocyclic compounds. The MIF(QM) maps and density(CSD) maps revealed that CH/π interaction formable areas are located above and below the aromatic ring of nitrogen-containing heterocyclic compounds, while CH/N interaction formable areas are positioned around the nitrogen atoms. Additionally, we confirmed again that the strengths of CH/π and CH/N interactions increase with the acidity of the donor CH groups.^43−47^ The average interaction energies of CH/π interactions are −1.1 and −1.8 kcal/mol for CH_4_ and benzene probes, respectively, while those for CH/N interactions are −0.6 and −1.3 kcal/mol, respectively. Additionally, the relative stability of CH/N interactions compared to CH/π interactions increases as the acidity of the donor CH group rises. The calculated MIF(QM) maps were validated by comparison with density(CSD) maps, which were, in turn, energetically explained by MIFs(QM). Additionally, it was confirmed that the MIF(QM, CH_3_) maps also aligned well with the density(PDB, alkyl CH) maps for the pyridine, pyrimidine, 1,3,5-triazine, quinoline, and indazole substructures. These results confirm that MIFs(QM) serve as a reliable computational tool for analyzing the 3D features of CH/π and CH/N interactions, especially when density(CSD) maps cannot be obtained due to limited crystal data. Furthermore, we obtained the approximation functions of MIFs(QM) through fitting calculations and confirmed that the MIFs(func) maps generated using these functions accurately reproduce MIFs(QM) maps. The MIF(func) calculations were successfully applied to analyze intermolecular interactions formed in the M102E/L99A mutant T4 lysozyme/pyridine and CDK2/indazole complexes. The MIFs(func) results suggest the formation of CH/π and CH/N interactions between the mutant T4 lysozyme and pyridine. Similarly, MIF(QM, CH_4_) highlights the potential CH/π interactions between CDK2 and indazole. The ligand-binding sites of T4 lysozyme and CDK2 exhibit favorable structures for binding pyridine and indazole, respectively, from the perspective of weak hydrogen bonds. The obtained approximation functions of MIFs(QM) hold significant promise for various applications, including analyses of protein/ligand interactions, construction of molecular libraries or databases, and the development of scoring functions for ligand docking calculations in future studies.

## Supporting Information Available

Table S1: Number of complex structures with ligands containing aromatic nitrogen-containing heterocycle substructures registered in PDB. Table S2: Detailed conditions of contact searches in the CSD. Figure S1: Relation between calculated MIF(QM, BZ) energies (not normalized) using M06-2X/aug-cc-pVDZ(CP) and M06-2X/6-31G(d,p)(CP) for pyridine. Figure S2: MIF(QM) maps of pyrimidine and density(CSD) maps where the central group is the pyrimidine substructure. Figure S3: Density(CSD) maps of the pyridine substructure. Figure S4: Density(CSD) maps of the pyrimidine substructure. Figures S5–S12: Comparisons between MIF(QM, CH_4_) and MIF(func, CH_4_) maps and between MIF(QM, BZ) and MIF(func, BZ) maps for pyrrole, pyrazole, imidazole, 1,2,3-triazole, pyridazine, pyrimidine, pyrazine, benzene, 1,3,5-triazine, 1,2,4-triazine, quinoline, indole, indazole, benzimidazole, and purine. Figure S13: Relations between MIF(QM, CH_4_) and MIF(func, CH_4_) energies and between MIF(QM, BZ) and MIF(func, BZ) energies for pyridine (PDF)
List of coefficients of approximation functions of MIFs(QM) (XLSX)
List of MIF(QM) and MIF(func) energies (XLSX)
Input files of the Gaussian16 program (ZIP)
List of PDB IDs of the structures used for the density calculations (XLSX)
