# Supplementary material for: Three-Dimensional CH/π and CH/N Interactions from Quantum-Mechanical and Database Analyses
Source: J Chem Inf Model. 2025 Apr 14;65(8):4116–27. doi: 10.1021/acs.jcim.5c00124 (PMC12042816; doi:10.1021/acs.jcim.5c00124)
Supplement: Supplementary file 1 — ci5c00124_si_001.pdf [file ci5c00124_si_001.pdf]

Supporting Information

Three-Dimensional CH/π and CH/N Interactions from Quantum-Mechanical and Database Analyses

Daichi Hayakawa, Hiroaki Gouda*

Division of Biophysical Chemistry, Department of Pharmaceutical Sciences,
Graduate School of Pharmacy, Showa University,
1-5-8 Hatanodai, Shinagawa-ku, Tokyo, 142-8555, Japan.

*d-hayakawa@pharm.showa-u.ac.jp

Table of contents

TABLE S1 **S3**

TABLE S2 **S4**

FIGURE S1 **S5**

FIGURE S2 **S6**

FIGURE S3 **S7**

FIGURE S4 **S8**

FIGURE S5 **S9**

FIGURE S6 **S10**

FIGURE S7 **S11**

FIGURE S8 **S12**

FIGURE S9 **S13**

FIGURE S10 **S14**

FIGURE S11 **S15**

FIGURE S12 **S16**

FIGURE S13 **S16**

Table S1. Number of complex structures with ligands containing aromatic nitrogen-containing heterocycle substructures registered in PDB.

Compounds	Exact mach[a]	Substructure[b]	Compounds	Exact mach[a]	Substructure[b]
purine	0	31,090	**pyrimidine**	1	43,597
indole	19	2,699	**imidazole**	998	35,759
quinoline	0	1,644	**pyridine**	3	15,172
benzimidazole	15	1,398	**Pyrrole**	0	13373
qinazoline	1	940	**pyrazole**	5	3,482
isoquinoline	4	761	**pyrazine**	0	1,321
indazole	9	536	**1H-1,2,3-triazole**	2	850
Indoline	2	526	**pyridazine**	0	540
pteridine	0	438	**1,3,5-triazine**	0	236
quinoxaline	0	376	**1,2,4-triazine**	0	138
3H-indole	0	237	1,2,4,5-tetrazine	0	0
phthalazine	2	98			
1,8-naphthyridine	1	53			
indolizine	0	36			
pyrrolizidine	0	33			
isoindole	0	32			
1,5-naphthyridine	0	26			
cinnoline	0	10			
quinolizine	0	0			

[a] The ligand molecule whose structure exactly agrees with the query structure.

[b] The ligand molecule that contains the substructure.

Table S2. Detailed conditions of contact searches in CSD.

Central group	Contact group	R-factor	Including powder	Number of fragments
Pyridine	X–CH_3	<= 0.05	Only single crystal	2498
	X=CH_2	All	Only single crystal	1134
	C_5X_5CH	<=0.05	Only single crystal	1395
	X≡CH	All	Including powder	852
Pyrimidine	X–CH_3	<= 0.05	Only single crystal	2418
	X=CH_2	All	Only single crystal	237
	C_5X_5CH	<=0.05	Only single crystal	1411
	X≡CH	All	Including powder	204

[a]The numbers of fragments used to generate scatter plots and density(CSD) maps.

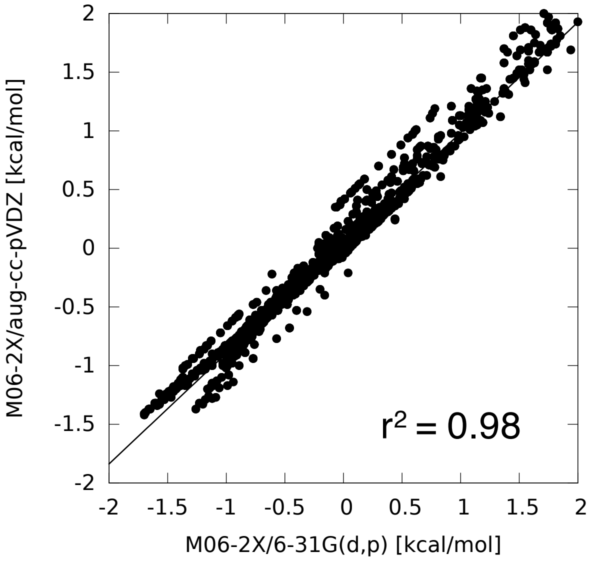

Figure S1. Relation between calculated MIF(QM, BZ) energies (not normalized) with M06-2X/aug-cc-pVDZ(CP) and M06-2X/6-31G(d,p)(CP) for pyridine.

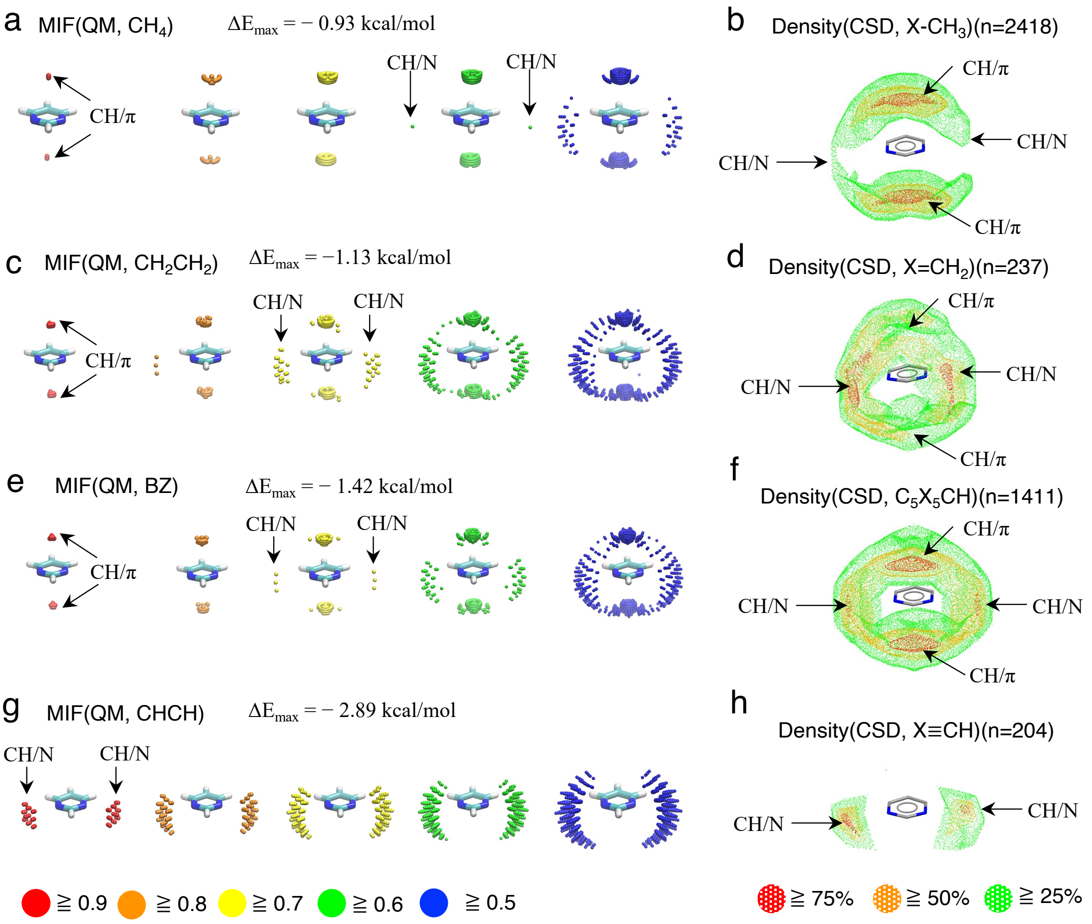

Figure S2. MIF(QM) maps of pyrimidine with (a) CH_4, (c) CH_2CH_2, (e) Benzene, and (g) CHCH probes. 3D variations in MIFs(QM) are depicted by spheres colored according to the MIF energy. MIFs(QM) were obtained by M06-2X/aug-cc-pVDZ calculations. Density(CSD) maps whose central group is pyrimidine substructure with (b) X–CH_3, (d) X=CH_2, (f) C_5X_5CH(f), and (h) X≡CH contact groups. The areas with densities of 75%, 50%, and 25% or higher of the maximum value are shown in red, orange, and green, respectively. (b) was drawn based on the positions of carbon atoms, and (d), (f), and (h) were drawn based on the positions of hydrogen atoms. All density(CSD) maps were obtained by CSD analyses using IsoGen and Isostar programs.

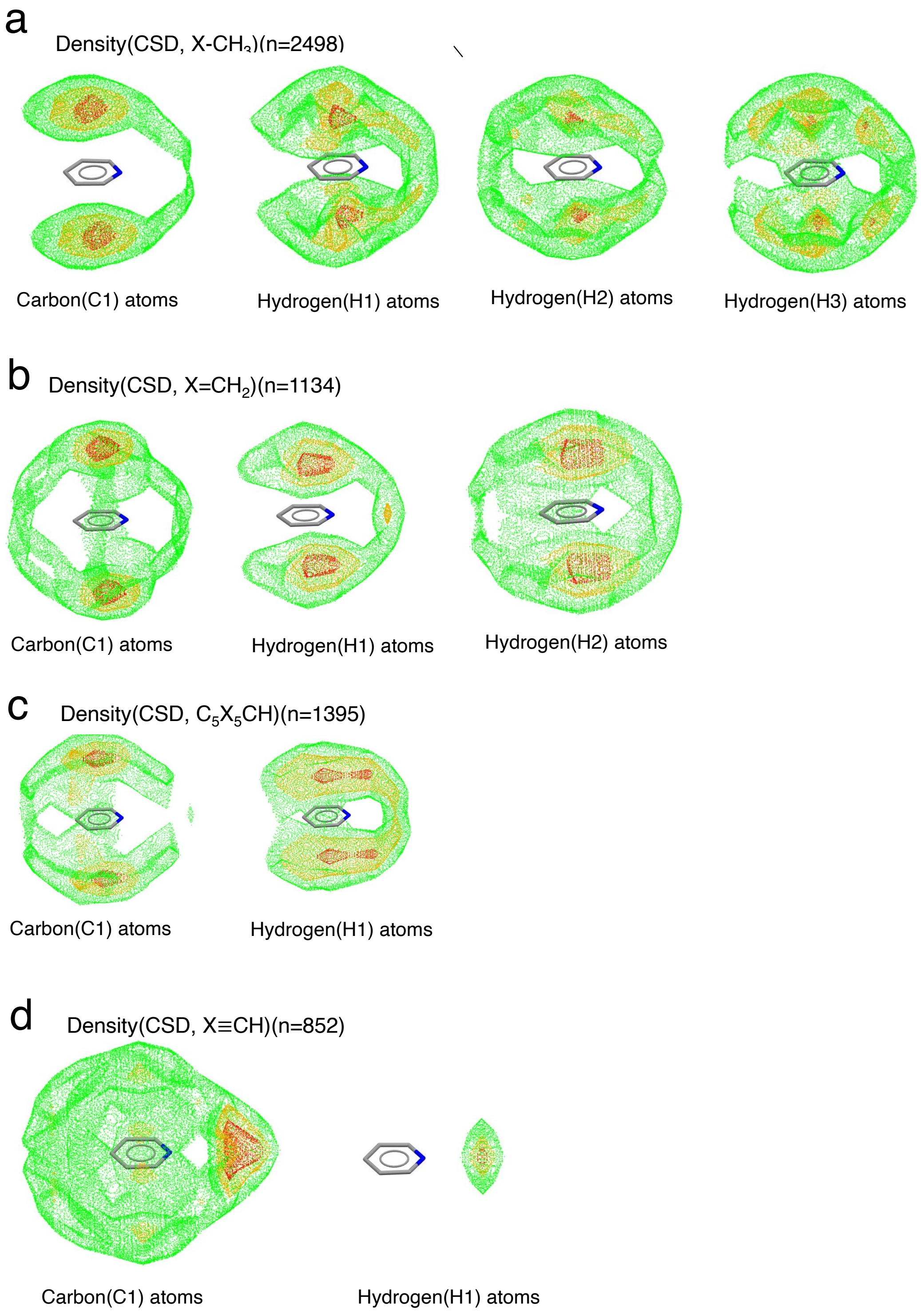

Figure S3. Density(CSD) maps of pyridine substructure with (a) X–CH_3, (b) X=CH_2, (c) C_5X_5CH, and (d) X≡CH contact groups. The atoms selected for the density calculation are marked in the figure. All density(CSD) maps were obtained by CSD analyses using IsoGen and Isostar programs.

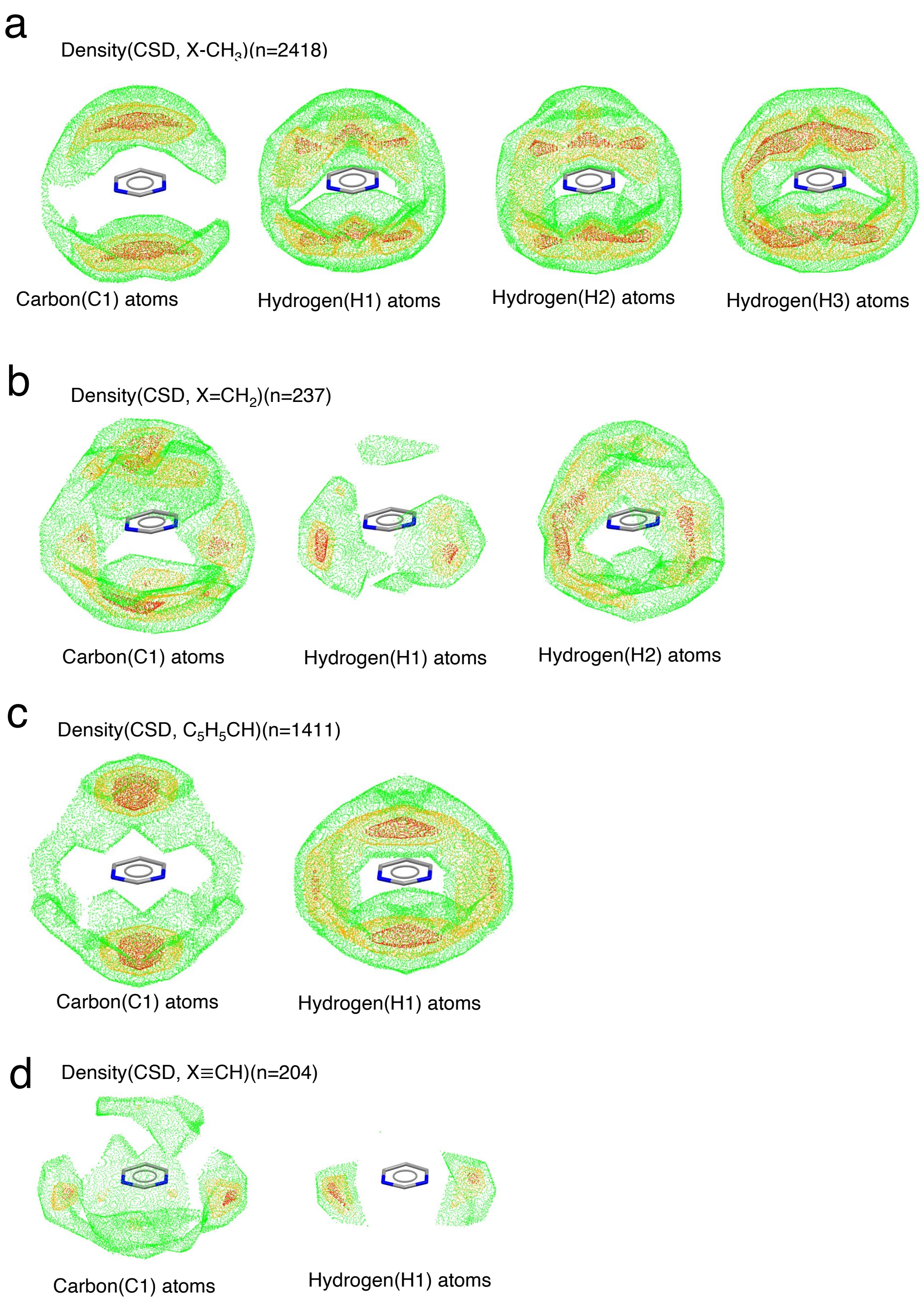

Figure S4. Density(CSD) maps of pyrimidine substructure with (a) X–CH_3, (b) X=CH_2, (c) C_5X_5CH, and (d) X≡CH contact groups. The atoms selected for the density calculation are marked in the figure. All density(CSD) maps were obtained by CSD analyses using IsoGen and Isostar programs.

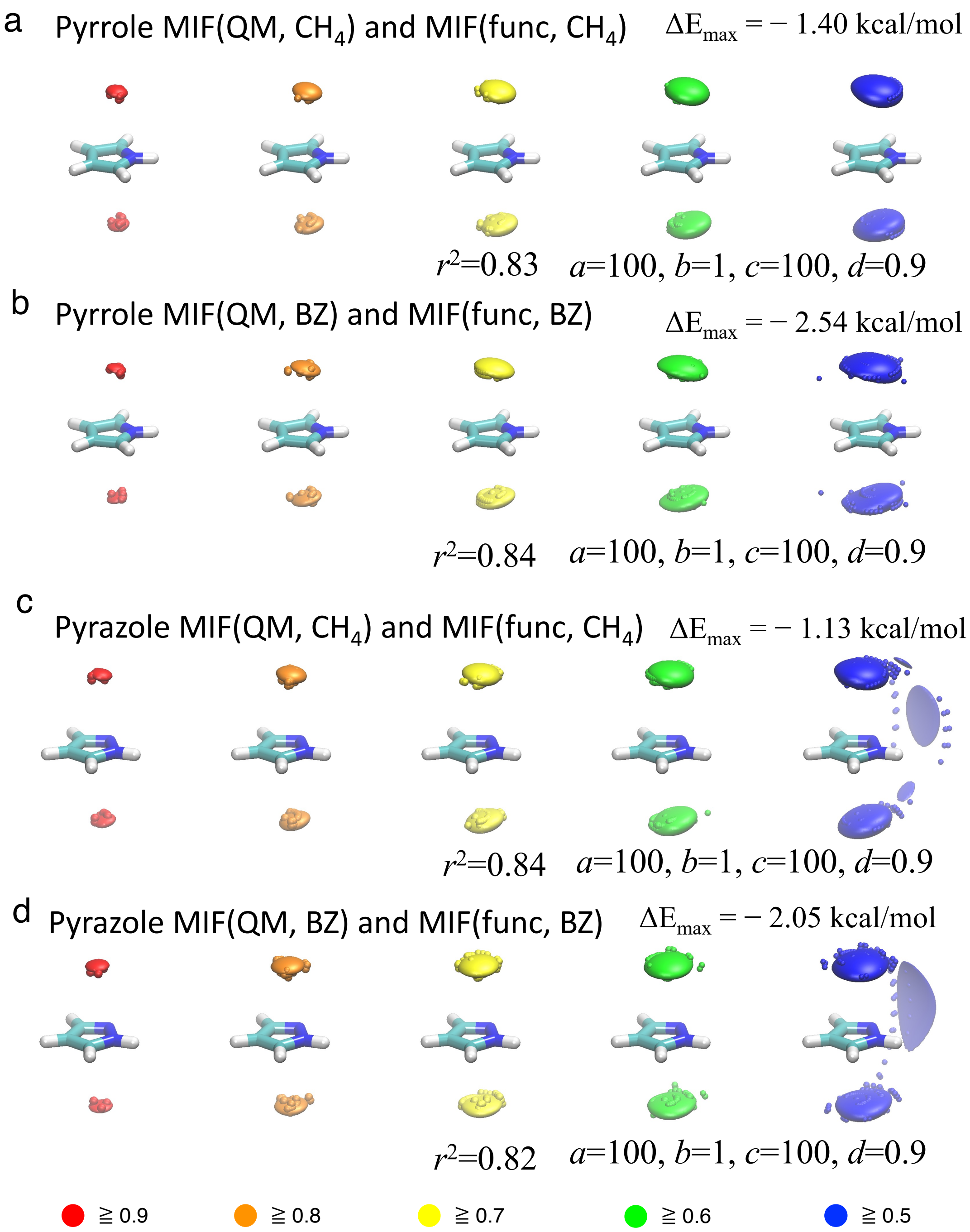

Figure S5. Superposition of (a) MIF(QM, CH_4) and MIF(func, CH_4) of pyrrole, (b) MIF(QM, BZ) and MIF(func, BZ) of pyrrole, (c) MIF(QM, CH_4) and MIF(func, CH_4) of pyrazole, and (d) MIF(QM, BZ) and MIF(func, BZ) of pyrazole. 3D variations in MIFs(QM) are depicted by spheres colored according to the MIF energy. MIFs(func) are described by surface representation with the same color key.

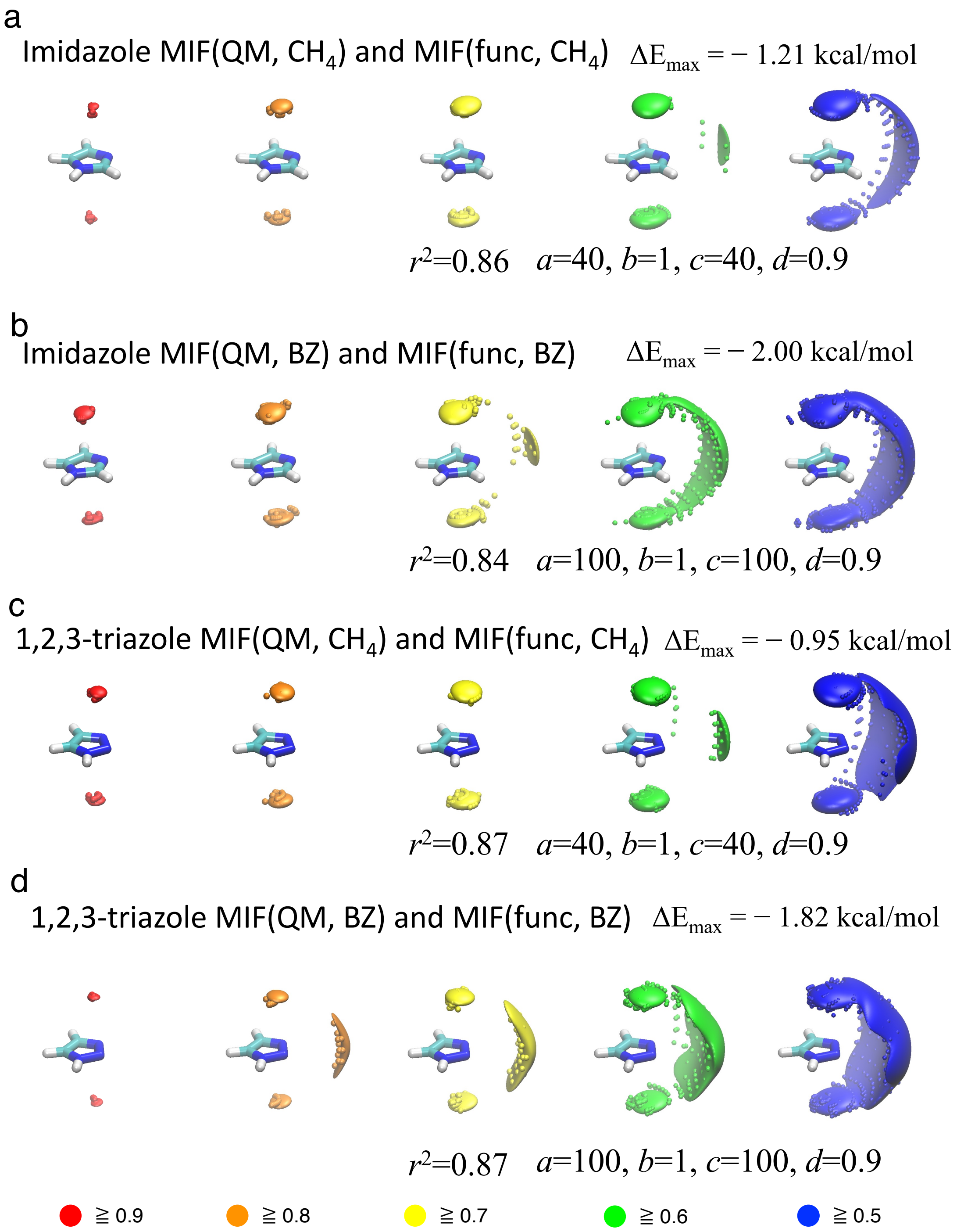

Figure S6. Superposition of (a) MIF(QM, CH_4) and MIF(func, CH_4) of imidazole, (b) MIF(QM, BZ) and MIF(func, BZ) of imidazole, (c) MIF(QM, CH_4) and MIF(func, CH_4) of 1,2,3-triazole, and (d) MIF(QM, BZ) and MIF(func, BZ) of 1,2,3-triazole. 3D variations in MIFs(QM) are depicted by spheres colored according to the MIF energy. MIFs(func) are described by surface representation with the same color key.

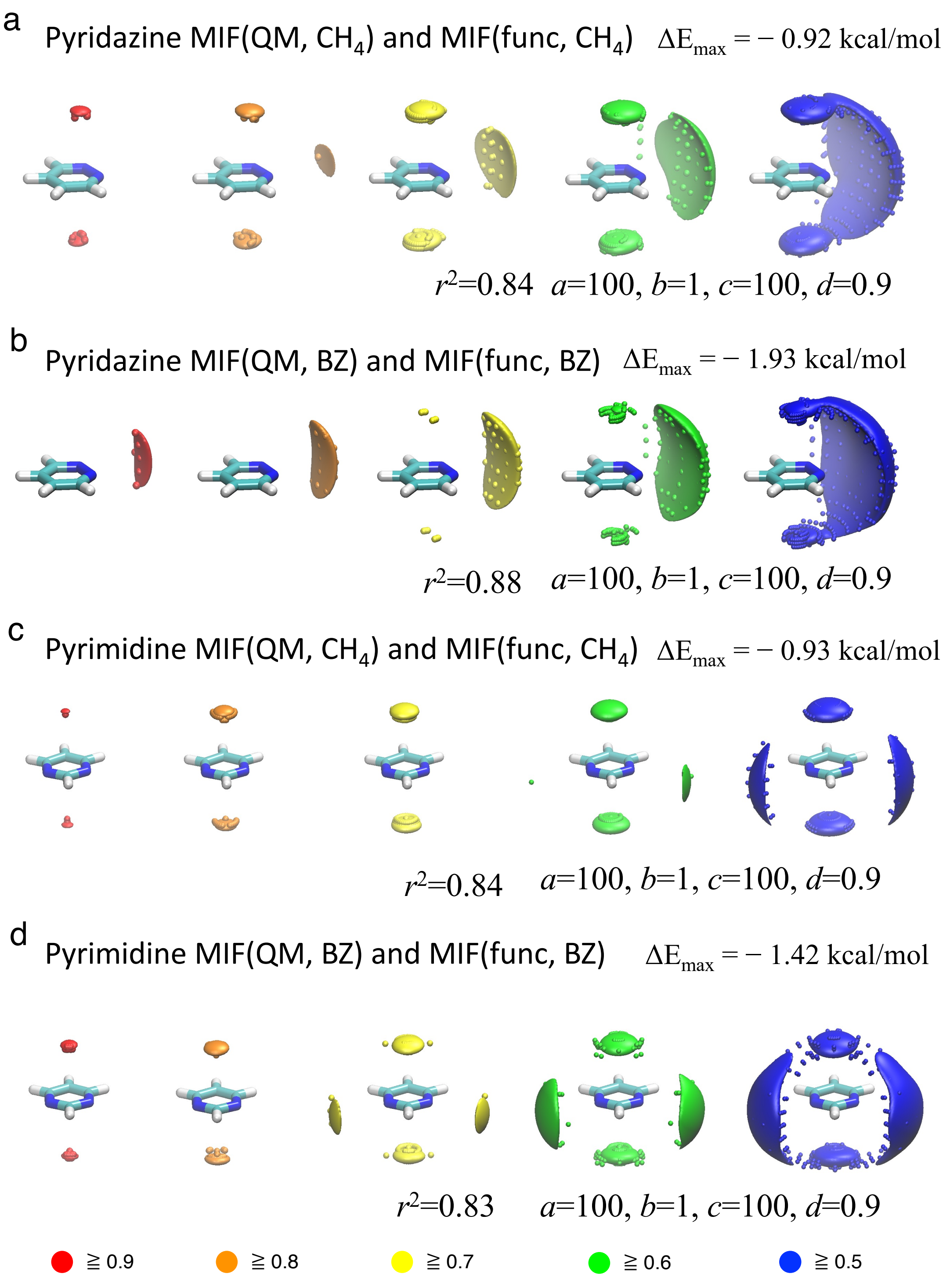

Figure S7. Superposition of (a) MIF(QM, CH_4) and MIF(func, CH_4) of pyridazine, (b) MIF(QM, BZ) and MIF(func, BZ) of pyridazine, (c) MIF(QM, CH_4) and MIF(func, CH_4) of pyrimidine, and (d) MIF(QM, BZ) and MIF(func, BZ) of pyrimidine. 3D variations in MIFs(QM) are depicted by spheres colored according to the MIF energy. MIFs(func) are described by surface representation with the same color key.

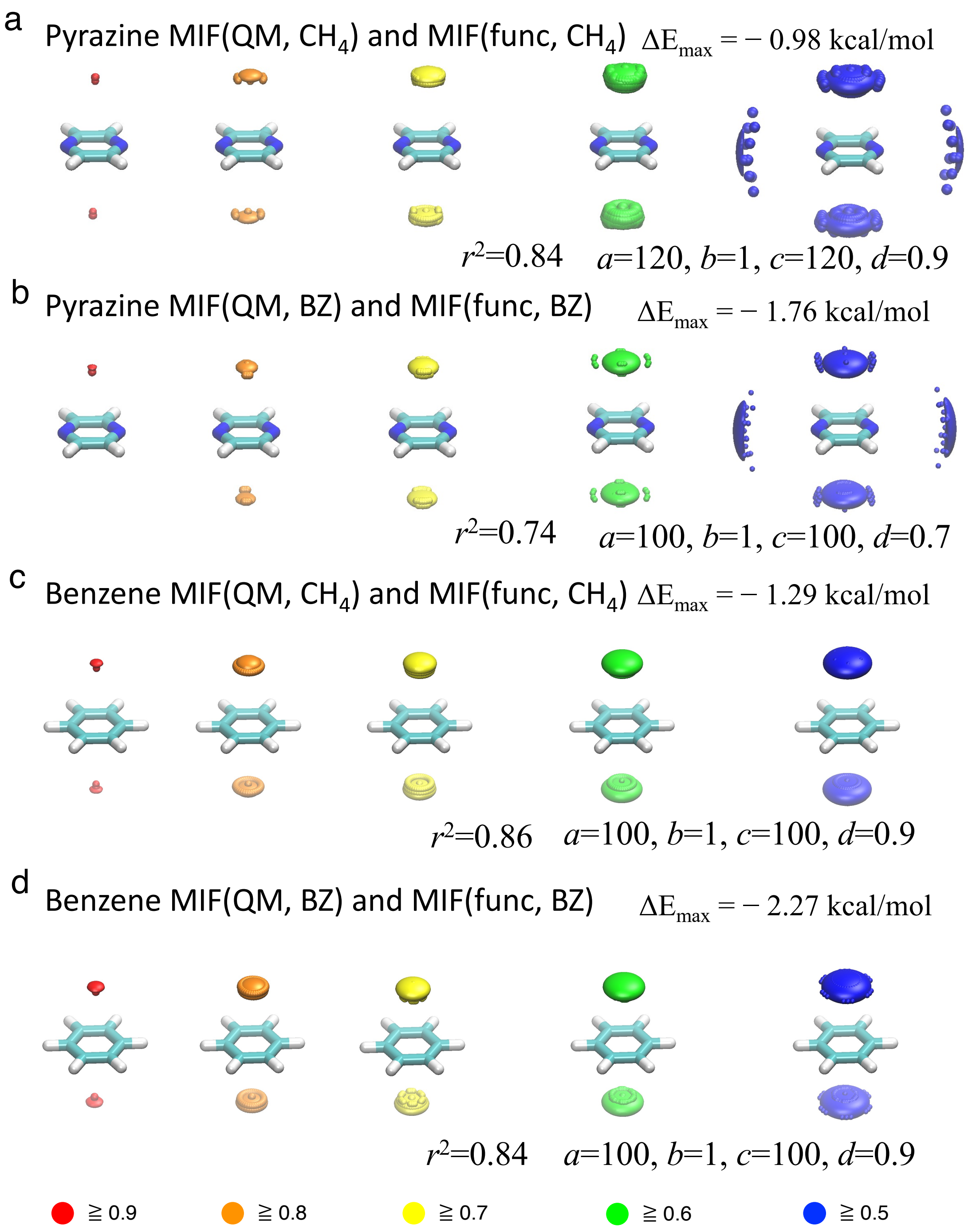

Figure S8. Superposition of (a) MIF(QM, CH_4) and MIF(func, CH_4) of pyrazine, (b) MIF(QM, BZ) and MIF(func, BZ) of pyrazine, (c) MIF(QM, CH_4) and MIF(func, CH_4) of benzene, and (d) MIF(QM, BZ) and MIF(func, BZ) of benzene. 3D variations in MIFs(QM) are depicted by spheres colored according to the MIF energy. MIFs(func) are described by surface representation with the same color key.

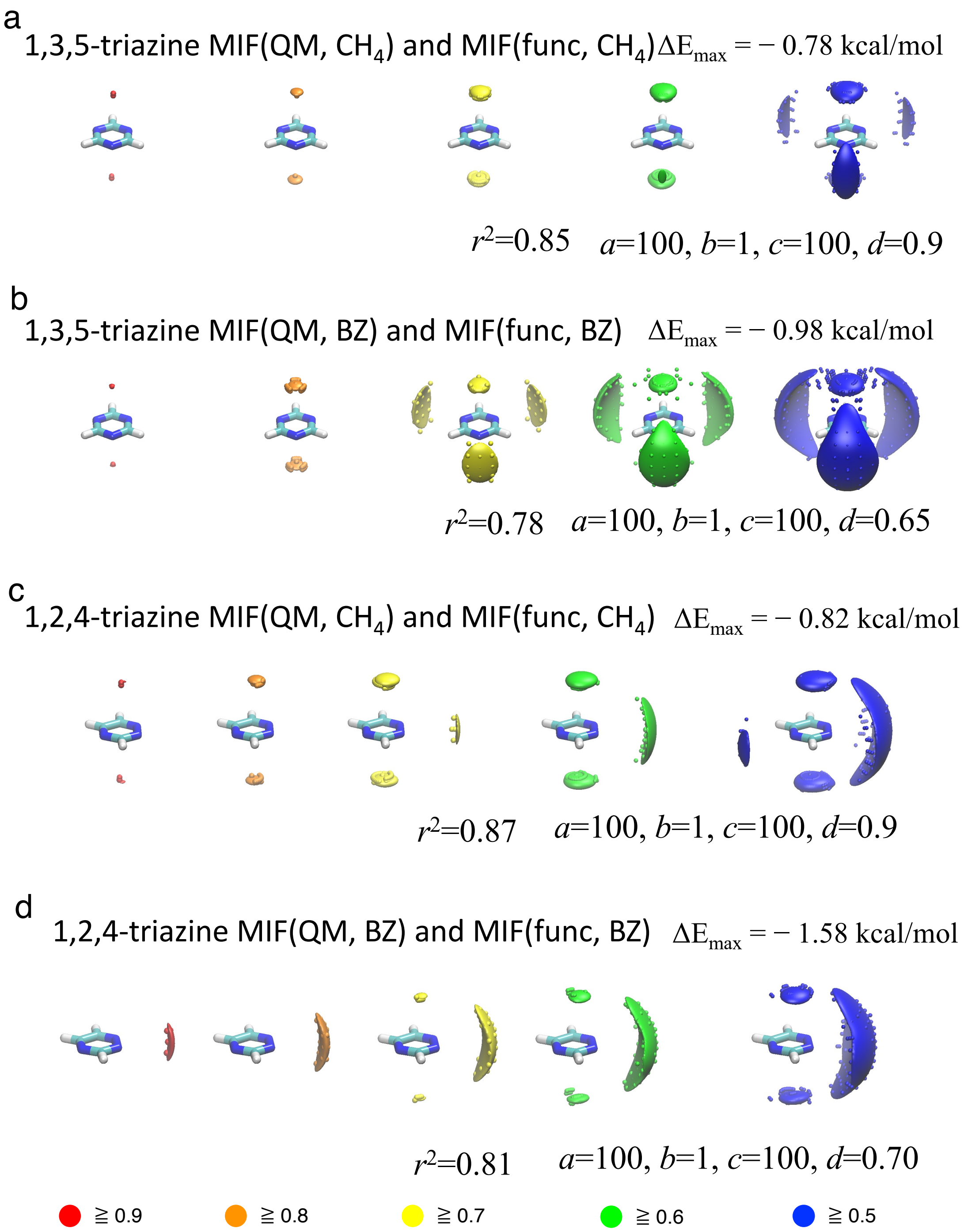

Figure S9. Superposition of (a) MIF(QM, CH_4) and MIF(func, CH_4) of 1,3,5-triazine, (b) MIF(QM, BZ) and MIF(func, BZ) of 1,3,5-triazine, (c) MIF(QM, CH_4) and MIF(func, CH_4) of 1,2,4-triazine, and (d) MIF(QM, BZ) and MIF(func, BZ) of 1,2,4-triazine. 3D variations in MIFs(QM) are depicted by spheres colored according to the MIF energy. MIFs(func) are described by surface representation with the same color key.

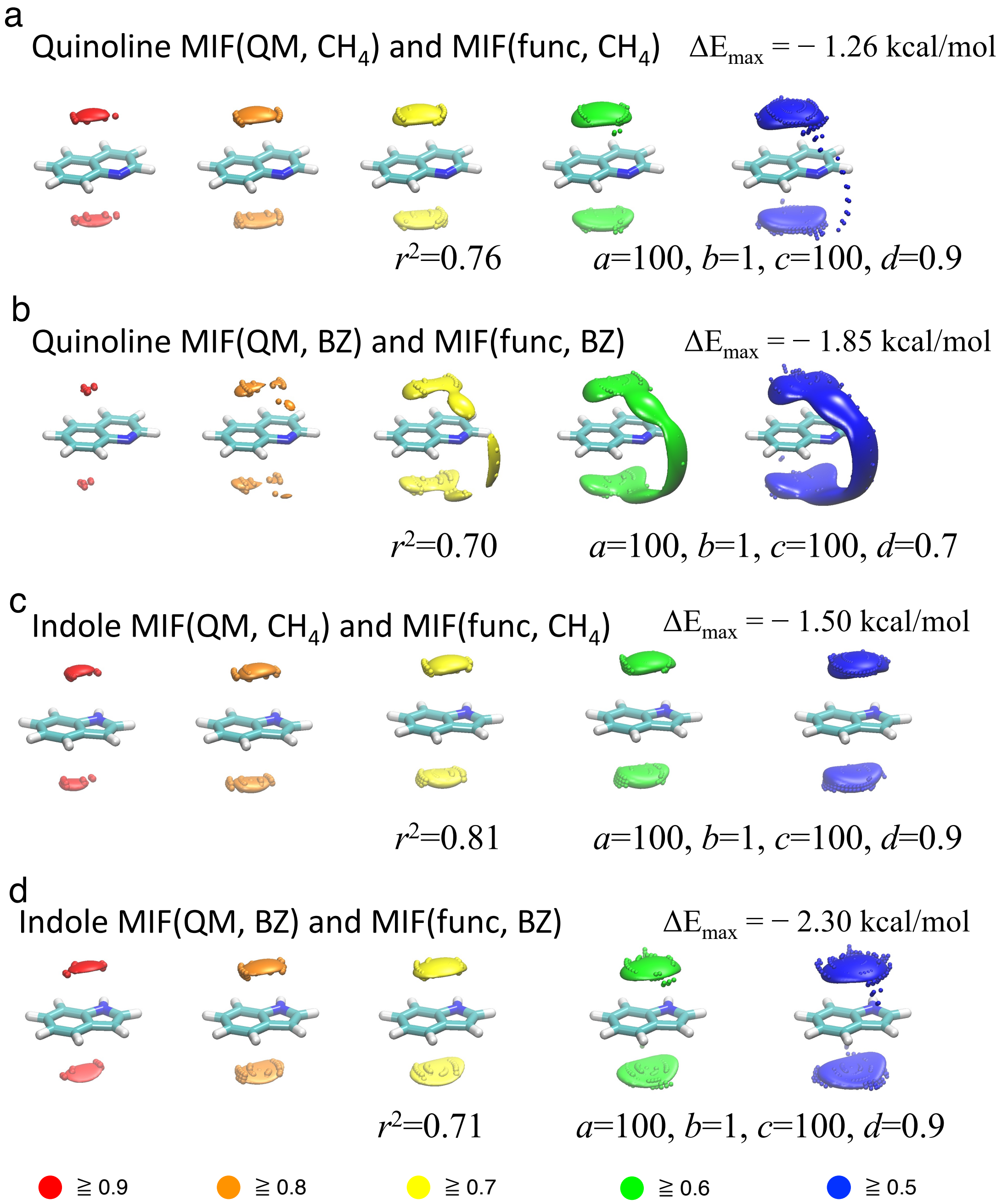

Figure S10. Superposition of (a) MIF(QM, CH_4) and MIF(func, CH_4) of quinoline, (b) MIF(QM, BZ) and MIF(func, BZ) of quinoline, (c) MIF(QM, CH_4) and MIF(func, CH_4) of indole, and (d) MIF(QM, BZ) and MIF(func, BZ) of indole. 3D variations in MIFs(QM) are depicted by spheres colored according to the MIF energy. MIFs(func) are described by surface representation with the same color key.

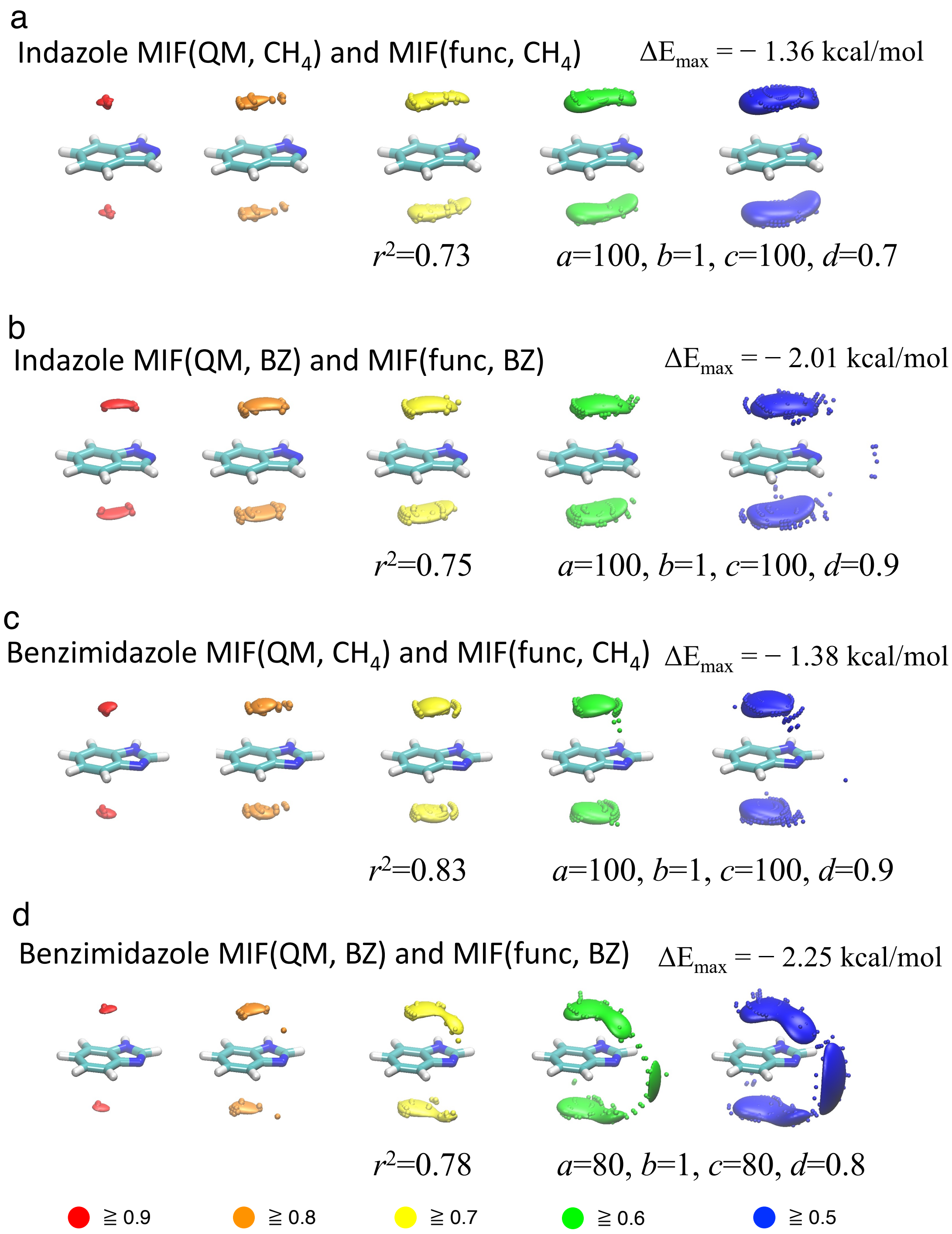

Figure S11. Superposition of (a) MIF(QM, CH_4) and MIF(func, CH_4) of indazole, (b) MIF(QM, BZ) and MIF(func, BZ) of indazole, (c) MIF(QM, CH_4) and MIF(func, CH_4) of benzimidazole, and (d) MIF(QM, BZ) and MIF(func, BZ) of benzimidazole. 3D variations in MIFs(QM) are depicted by spheres colored according to the MIF energy. MIFs(func) are described by surface representation with the same color key.

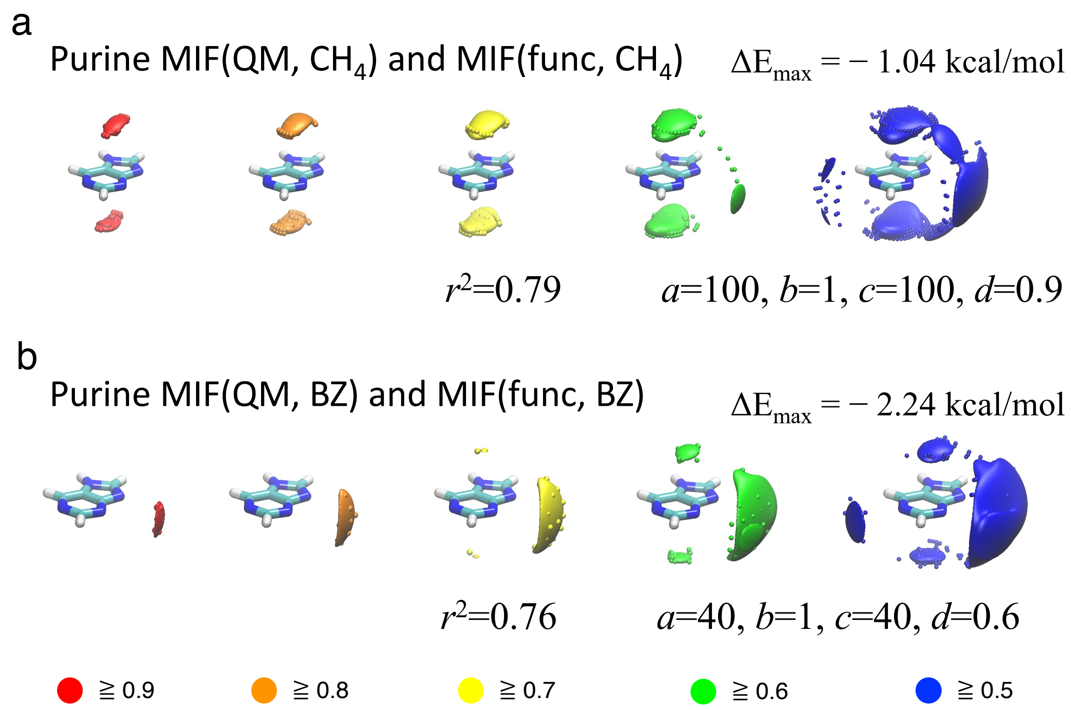

Figure S12. Superposition (a) of MIF(QM, CH_4) and MIF(func, CH_4) and (b) of MIF(QM, BZ) and MIF(func, BZ) of purine. 3D variations in MIFs(QM) are depicted by spheres colored according to the MIF energy. MIFs(func) are described by surface representation with the same color key.

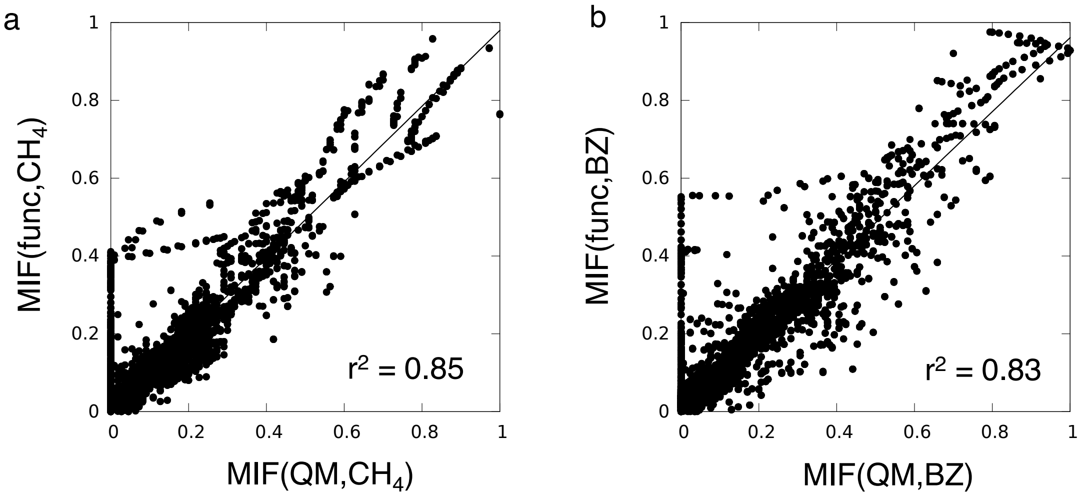

Figure S13. Relations between (a) MIF(QM, CH_4) and MIF(func, CH_4) energies and (b) between MIF(QM, BZ) and MIF(func, BZ) energies for pyridine.
